# Design, Synthesis and Molecular Docking Study of Novel 3-Phenyl-β-Alanine-Based Oxadiazole Analogues as Potent Carbonic Anhydrase II Inhibitors

**DOI:** 10.3390/molecules27030816

**Published:** 2022-01-26

**Authors:** Kashif Rafiq, Najeeb Ur Rehman, Sobia Ahsan Halim, Majid Khan, Ajmal Khan, Ahmed Al-Harrasi

**Affiliations:** 1 Natural and Medical Sciences Research Center, University of Nizwa, P.O Box 33, Birkat Al Mauz, Nizwa 616, Oman; kashifrafiq@unizwa.edu.om (K.R.); sobia_halim@unizwa.edu.om (S.A.H.); majidk166@yahoo.com (M.K.); ajmalkhan@unizwa.edu.om (A.K.); 2 Department of Chemistry, Abdul Wali Khan University Mardan, Mardan 23200, Pakistan; 3 H. E. J. Research Institute of Chemistry, International Center for Chemical and Biological Sciences, University of Karachi, Karachi 75270, Pakistan

**Keywords:** 3-phenyl-β-alanine 1,3,4-oxadiazole hybrids, carbonic anhydrase-II, α-glucosidase, structure-activity relationship, molecular docking

## Abstract

Carbonic anhydrase-II (CA-II) is strongly related with gastric, glaucoma, tumors, malignant brain, renal and pancreatic carcinomas and is mainly involved in the regulation of the bicarbonate concentration in the eyes. With an aim to develop novel heterocyclic hybrids as potent enzyme inhibitors, we synthesized a series of twelve novel 3-phenyl-β-alanine 1,3,4-oxadiazole hybrids (**4a**–**l**), characterized by ^1^H- and ^13^C-NMR with the support of HRESIMS, and evaluated for their inhibitory activity against CA-II. The CA-II inhibition results clearly indicated that the 3-phenyl-β-alanine 1,3,4-oxadiazole derivatives **4a**–**l** exhibited selective inhibition against CA-II. All the compounds (except **4d**) exhibited good to moderate CA-II inhibitory activities with IC_50_ value in range of 12.1 to 53.6 µM. Among all the compounds, **4a** (12.1 ± 0.86 µM), **4c** (13.8 ± 0.64 µM), **4b** (19.1 ± 0.88 µM) and **4h** (20.7 ± 1.13 µM) are the most active hybrids against carbonic CA-II. Moreover, molecular docking was performed to understand the putative binding mode of the active compounds. The docking results indicates that these compounds block the biological activity of CA-II by nicely fitting at the entrance of the active site of CA-II. These compounds specifically mediating hydrogen bonding with Thr199, Thr200, Gln92 of CA-II.

## 1. Introduction

Carbonic anhydrases (CAs, EC 4.2.1.1) are a class of well-studied metalloenzymes widely distributed in living organisms [1]. These are strongly involved in regulating cell homeostasis, intracellular pH, fluid secretion, ion transport and biosynthetic reactions by catalyzing the reversible hydration of carbon dioxide (CO_2_) to bicarbonate ion (HCO^3−^) and proton (H^+^) [2,3,4,5,6,7]. This simple reaction is crucial for many physiological mechanisms including electrolyte secretion, acid-base tuning, tumorigenesis, respiration, bone resorption, calcification and biosynthesis of important molecules such as glucose, urea, and lipids [8,9]. These enzymes are common in almost all organisms from simple to complex [10,11]. The extracellular pH in tumors is more acidic than intracellular pH [11]. To generate the pH gradient between the extracellular and intracellular compartments, tumor cells express ion transport proteins and CA enzymes [12,13,14]. The CA-II is expressed in malignant brain tumors [15,16,17], renal cancer cell lines, and gastric and pancreatic carcinomas [15,17]. CA-II has also been used since long time for the treatment of glaucoma, epilepsy, leukemia, and cystic fibrosis [18,19]. It is remarkable that ubiquitous hCA-I and II are the main off-target isoforms because these are involved in many physiological and biochemical processes [20]. Due to the key role of this enzyme in several diseases, its inhibition is considered therapeutically important.

**Figure 1:**
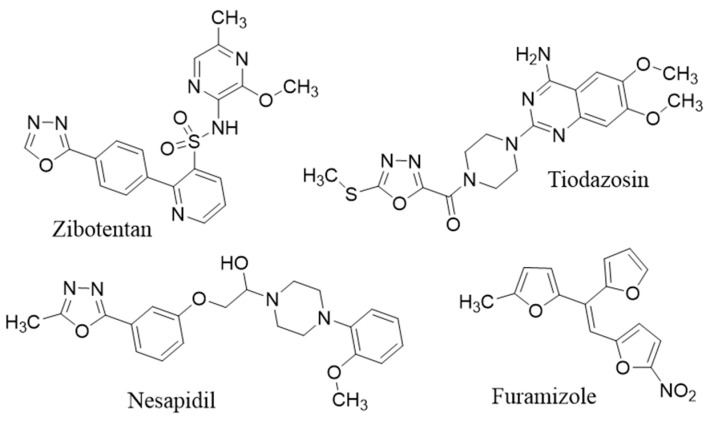
Some reported 1,3,4-oxadiazole derivative drugs.

Oxadiazoles are heterocyclic compounds which have a diversity of useful biological effects including antibacterial [21], antifungal [22], analgesic [23], anti-inflammatory [24], antiviral [25], anticancer [26], antihypertensive [27], anticonvulsant [28] and anti-diabetic [29]. 1,3,4-Oxadiazole nucleus is present in the molecules of drugs such as: furamizole (antibacterial), tiodazosin (an α-1 adrenergic antagonist), nesapidil (antihypertensive), raltegravir (antiretroviral), and zibotentan (anticancer) (Figure 1) [30]. The most commonly used synthetic route for the synthesis of 1,3,4-oxadiazoles includes the reactions of acid hydrazides with acid chlorides, carboxaldehyde, carboxylic acids and cyclization of the formed acylhydrazines using a dehydrating agent, such as phosphorous pentaoxide, thionyl chloride, or phosphorous oxychloride [31]. On the other hand, 1,3,4-oxadiazoles are thermally stable and neutral heteroaromatic compounds containing two nitrogens and one oxygen atom, affects the pharmacokinetic and physicochemical properties of the compounds in which it is present [32]. It makes diverse noncovalent interactions with various active sites of enzymes and receptors in biological systems and, thus display versatile pharmacological activities like anti-inflammatory [33], antidepressant [34], anti-proliferative [35], analgesic [36] and antiviral effect [32,37,38].

Previously, Vats et al. have synthesized novel 4-functionalized 1,5-diaryl-1,2,3-triazoles containing benzenesulfonamide moiety as carbonic anhydrase I, II, IV and IX inhibitors [39], while novel benzenesulfonamides bearing 1,2,3-triazole linked hydroxy trifluoromethylpyrazolines and hydrazones as selective carbonic anhydrase isoforms IX and XII inhibitors were reported by Sharma et al. [40]. Similarly, Kumar et al. described that the synthesis of novel benzenesulfonamide containing 1,2,3-triazoles [41] and benzenesulphonamide-bearing 1,4,5-trisubstituted-1,2,3-triazoles [42] showed potent inhibition against human carbonic anhydrase isoforms I, II, IV and IX inhibitors. Bianco et al. have successfully been synthesized N-acylbenzenesulphonamide dihydro-1,3,4-oxadiazole hybrids against hCA IX and XII [43]. Recently, Sharma et al. have reported the novel benzenesulfonamides incorporating 1,3,4-oxadiazole hybrids as potent inhibitor of carbonic anhydrase I, II, IX, and XII isoenzymes [44], while Swain et al., have efficiently been synthesized have efficiently been synthesized benzenesulphonamide based 1,3,4-oxadiazoles as selective carbonic anhydrase XIII inhibitors [45]. Our group recently reported a novel class of CAs inhibitors belonging to the 1H-1,2,3-triazole derivatives [46,47].

Our focus was on the identification of novel drug-like compounds against CA-II enzyme to combat CA-II related disorders. Keeping in mind the importance of these scaffolds in the present work, we designed and synthesized a series of novel 3-phenyl-β-alanine 1,3,4-oxadiazole hybrids **4a**–**l** with the oxadiazole ring offering an important pharmacophore. With the hope to obtain an effective carbonic anhydrase II enzyme inhibitors, we planned to synthesize new compounds, characterized by spectroscopic techniques including ^1^H-, ^13^C-NMR and HRMS, and to explore their carbonic anhydrase enzyme inhibition. Later, computational docking method was applied to investigate the mode of binding of these compounds in the active site of CA-II.

## 2. Results and Discussion

### 2.1. Chemistry

The interest in finding an effective carbonic anhydrase II enzyme inhibitor has been increased in recent decades, especially with the exploring of possible relationships between carbonic anhydrase II and cancer [48,49,50]. CA is present in human (h) with sixteen (16) different isoforms identified from hCA I-hCA XV. All these isoforms are widely dispersed in different tissues/organs and are associated with a range of pivotal physiological activities. Due to their involvement in various physiological roles, inhibitors of different human isoforms of CA have found clinical applications for the treatment of various diseases including glaucoma, retinopathy, epilepsy, hemolytic anemia, and obesity [51]. However, clinically used inhibitors of CA (acetazolamide, brinzolamide, dorzolamide, etc.) are not selective causing the undesirable side effects. Recently we have investigated the interaction of CA-II isozymes with several types of natural and synthetic compounds [46,47,52,53]. Furthermore, 1,3,4-oxadiazole derivatives were found to be strong inhibitors against carbonic anhydrase II enzyme [39,40,41,42,44]. Inspired by these advances, we sought to investigate the application of this reagent in oxadiazole synthesis.

**Scheme 1:**
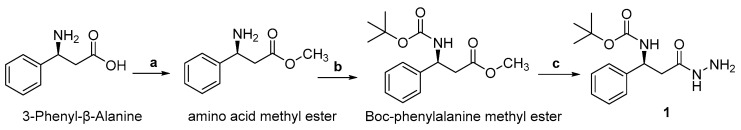
Reagents and conditions: (**a**) SOCl_2_, MeOH, 0 °C, overnight; (**b**) NaCO_3_, H_2_O, Boc, EtOAc (**c**) NH_2_NH_2_.H_2_O, MeOH, rt, 24 h (**1**).

Molecular iodine plays an important role in organic synthesis, owing to its commercial availability, low cost, and low toxicity [54,55]. Recently, it has been successfully employed to synthesize indole derivatives [56,57] and oxazoles [58,59,60]. Compound **1** was synthesized through esterification of 3-phenyl-β-alanine in methanol (MeOH) by adding thionyl chloride, followed by protection of the amino group as tert-butyloxycarbonyl (Boc), which is then treated with hydrazine in the presence of MeOH at room temperature Scheme 1).

**Scheme 2:**
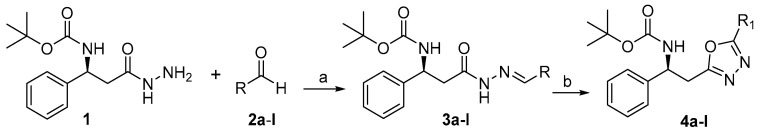
Reagents and conditions: (**a**) EtOH, 80 °C, Reflux overnight, (**3a**–**l**, 90%); (**b**) K_2_CO_3_, DMSO, 100 °C, Reflux overnight (**4a**–**l**).

Our investigation started with the cyclization of 3-phenyl-β-alanine hydrazones **3a**–**l** to the corresponding 1,3,4-oxadiazole **4a**–**l**. The synthesis and NMR data of the substrates **3a**–**l** were already reported by our group. These compounds were prepared via the condensation of different benzaldehyde moieties **2a**–**l** (0.6 mmol, 1.2 equiv.) with phenylalanine hydrazide (**1**, 0.5 mmol, 1 equiv.) in ethanol at refluxing temperature in 90% yield [52] (Scheme 2). The oxidative cyclization of **3a**–**l** to **4a**–**l** was achieved by utilizing molecular iodine in the presence of potassium carbonate (1.8 mmol, 3 equiv.) and DMSO (2 mL) which is the most effective media (solvent) for this conversion at 100 °C. The structures of the resulting compounds were confirmed by ^1^H-, IR, and ^13^C-NMR spectroscopy as well as mass spectrometry (HRESIMS).

### 2.2. Carbonic Anhydrase-II Enzyme Inhibition and Structural-Activity Relationship

All the synthetic compounds reported herein, and standard carbonic anhydrase inhibitor acetazolamide were assessed for their inhibition properties towards the relevant recombinant human carbonic anhydrase-II using the colorimetric method. Most of the synthesized compounds strongly inhibited CA-II enzyme ranging from 12.1 to 53.6 μM. Among all, compounds **4a** (12.1 ± 0.86 μM), **4c** (13.8 ± 0.64 μM), **4i** (18.1 ± 1.31 μM), **4b** (19.1 ± 0.88 μM) and **4h** (20.7 ± 1.13 μM) were found to be the best CA-II inhibitors, while compounds **4g** (21.5 ± 0.99 μM), **4k** (22.4 ± 1.32 μM), **4f** (25.1 ± 1.04 μM), and **4e** (26.6 ± 0.80 μM) demonstrated moderate activity (Table 1). Compared to standard (acetazolamide), compound **4l** (53.6 ± 0.96 μM) was found to be weak inhibitor against CA-II among all derivatives. 

Comparing compound **4i** with **4b**, the higher activity of the **4i** may be possible due to the presence of -OH group at para position of phenyl ring. A comparison between 4g with **4h** revealed a slight decrease in inhibition of **4g**, which is likely due to the presence of nitro group (electron-withdrawing substituent) at ortho position of the phenyl ring. This suggests that the inhibitory activity of CA-II enzyme could be possibly increased with an electron-withdrawing substituent at para position of the phenyl ring. A slight change in activities between **4j** and **4k** as well as **4e** and **4f** was observed. The higher inhibition of **4k** and **4f** could be accounted for the arrangements of functional groups attached to the phenyl ring. Compound **4a** (IC_50_ = 12.1 μM) exerted a stronger inhibition than other compounds in the series, which is possibly due to the presence of pyridine ring instead of phenyl. Similarly, a higher inhibition of compound **4c** is observed when compared with compounds **4d** and **4l** which is probably due to the absence of electron withdrawing groups at para position of the phenyl ring. 

### 2.3. Molecular Docking Results

**Figure 2:**
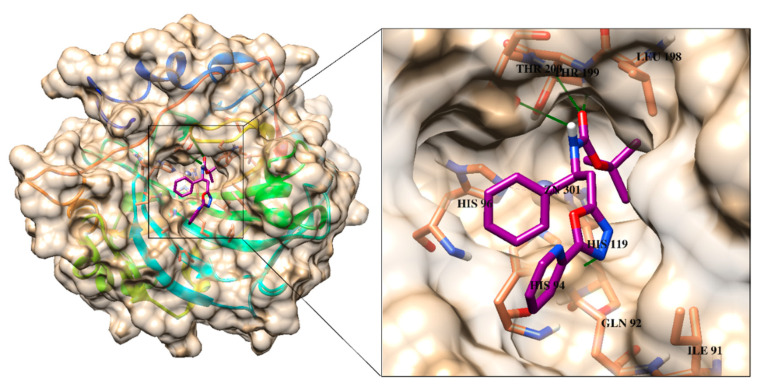
The binding mode of most active compound **4a** is shown in the active site of CA-II. The ligand is presented in magenta stick model, 3D-structure of CA-II is shown in surface model, interacting residues are depicted in coral stick model and H-bonds are presented in green lines.

All the active compounds were docked at the active site of CA-II (PDB code: 3HS4) to predict the best possible binding modes of each active compound through molecular docking. The standard drug, acetazolamide binds at the active site and mediates several interactions including ionic interactions with Zn ion, hydrogen bonding with the side chains of His94, His96, Thr199 and Thr200 within the active pocket of CA-II. The binding mode of acetazolamide is shown in Figure 2. Several active compounds, including **4a**, **4c** and **4i**, exhibited CA-II inhibitory activity (in range of 12.1 to 18.1 µM) higher than the standard drug. The binding mode of the most active compound **4a** depict that the carbamate moiety of the compound is involved in binding with the active site residues. Whereas oxadiazol ring and its substituted R group (pyridinyl ring) resided at the entrance of the active site. Moreover, the carbamate substituted phenyl ring was also fitted neatly in the groove near the active site entrance. The carbamate moiety of the compound mediated multiple H-bonding interaction with the amino group of Thr199 and Thr200, and side chain of Thr200. Moreover, the side chain of Gln92 also provides H-bond to the oxadiazole ring of the compound. We observed that the compound does not interact with the Zn ion in the active site, however, by interacting with several active site residues through H-bonds, and complete blockage of active site entrance, **4a** inhibits the function of CA-II. Similar orientation was observed for compound **4c** (the second highest active inhibitor), **4i** and **4b**, however, carbamate moiety of these compounds interacted with the amino group and the side chain of Thr200. Additionally, -OH group at the substituted hydroxyl phenyl ring of **4b** mediated H-bonding with the side chain of Gln92. The docked conformation of **4h** was completely like the binding mode of **4b**, the carbonyl oxygen of **4h** binds with the amino group of Thr200, however, the oxadiazol and the substituted nitrophenyl rings did not interact with the surrounding residues. Interestingly, the carbamate and the oxadiazol ring of **4g** do not interact with the surrounding residues, while its methoxy oxygen interacted with the amino group of Thr199 through H-bond. The substituted nitrophenyl rings of compounds **4h** and **4g** were found to be surface exposed. Similarly, the carbamate oxygen of **4k** and **4f** and oxadiazol ring of **4f** formed H-bonds with the amino nitrogen of Thr200, and side chain of Gln92, respectively. The binding mode of compounds **4e** and **4j** demonstrated that the carbamate nitrogen of **4e** interacted with a water molecule in the vicinity of active site, while the substituted R group mediated π-π interaction with the phenyl ring of Phe131, whereas the carbonyl oxygen of **4j** accepted H-bond with the amino group of Thr199. The least active compound of this series, **4l** only mediates bidentate interaction with the amino nitrogen and side chain -OH of Thr200 through its carbamate moiety. The binding interactions of each compound within the active site of CA-II are tabulated in Table 2. The docked view of the most active compound **4a** is presented in Figure 2.

The pharmacokinetic properties of the active compounds were predicted through BOILED- Egg model of SwissADME [61]. In the BOILED-Egg analysis, all the compounds showed high gastro-intestinal absorption, while none of the compounds exhibited blood brain barrier permeability. Moreover, all the compounds followed Lipinski rule of five drug-likeness criteria, and no PAIN alerts. In addition, accept **4a**, all the compounds were found as non-substrate for P-glycoprotein (Table 3). The predicted ADMET profile of the compounds suggest that these compounds could act as beneficial inhibitor of CA-II.

## 3. Material and Methods

### 3.1. General Instrumentation

All reagents were purchased from Sigma-Aldrich Chemical Company (St. Louis, MO, USA). Solvents used for chemical reactions were purified and dried by standard procedures. Melting point was determined using digital melting point apparatus SMP10 (Stuart^TM^, Cole-Parmer, Beacon Rd, Stone, Staffordshire, ST15 OSA, UK). Infrared (IR) spectra were recorded on an ATR-Tensor 37 attenuated total reflectance spectrometer (Bruker, Ettlingen, Baden-Württemberg, Germany) in the range from 400 to 4000 cm^−1^. High-resolution electrospray ionization mass spectrometry (HR-ESI-MS, Agilent technologies, 6530, Q-TOF LC/MS, Agilent, country of origin USA/EU, made in Singapore) was used for the determination of compound masses. The ^1^H- (600 MHz) and ^13^C- (150 MHz) NMR spectra were recorded on Bruker (Zürich, Switzerland) nuclear magnetic resonance spectrometers using the solvent peak as internal reference (CDCl_3_, δH: 7.26; δC: 77.2–76.8; DMSO, δH: 2.49; δC: 40.0–39.1). The following abbreviations were used to explain for NMR signals as s = singlet, d = doublet, dd = doublet of doublet, t = triplet, m = multiplet, *J* = coupling constant. Chemical shifts are expressed in parts per million (δ) values and coupling constants (*J*) are given in Hertz (Hz). All reactions were monitored by Thin-Layer-Chromatography (TLC, Merck, Darmstadt, Germany) using pre-coated aluminum sheets (silica gel 60 F_254_). TLC plates were visualized under the UV light at 254 and 366 nm and by spraying with the ninhydrin reagent. Solvents for chromatography were of technical grade and distilled prior to use.

### 3.2. General Procedure for the Synthesis 3-phenyl-β-alanine-1,3,4-Oxadiazoles Derivatives

3-Phenyl-β-alanine (10 mmol, 1.65 g) was used as a starting material for the synthesis of Boc-3-phenyl-β-alanine hydrazide (**1**). Esterification of the carboxylate group was done by the slow addition of thionyl chloride (15 mmol, 2.1 mL) to a solution of methanol (20 mL) containing 3-phenyl-β-alanine at 0 °C. The reaction solution was stirred for overnight (24 h.) at room temperature, while the completion of the reaction was carefully monitored by TLC for ensuring that all amount of amino acid changed to ester. After reaction completion, the solvent (MeOH) was evaporated under vacuum and the resultant product was filtered.

In the second step, amino acid methyl ester (9.8 mmol, 1.75 g) was added to a solution of anhydrous Na₂CO₃ (1.2 g, 12 mmol) and H_2_O (40 mL) in a 100 mL RB flask. Then, di-*tert*-butyl dicarbonate (Boc anhydride, 10 mmol, 2.1 g) was dissolved in EtoAc (20 mL), added to that solution and the reaction was allowed to stir for 24 h at room temperature. The progress of the reaction was continuously observed by TLC. When the reaction was completed, the pH was adjusted to 5.5 with oxalic acid and then the desired product was extracted with an organic solvent (EtOAc). The solvent was dried over anhydrous MgSO_4_ and evaporated under reduced pressure to obtain the corresponding Boc-phenylalanine methyl ester (91%). 

In the third step, hydrazine monohydrate (2 mL, 32 mmol) was added to a solution of methanol (20 mL) containing Boc-phenylalanine methyl ester (9.1 mmol, 2.5 g) and stirred under room temperature for 24 h to afford phenylalanine hydrazide (**1**). The reaction mixture was thoroughly checked by TLC. After reaction completion, the solvent was evaporated under reduced pressure and the product tert-butyl (S)-(3-hydrazinyl-3-oxo-1-phenylpropyl) carbamate (**1,** yield 89%) was washed with methanol. White powder; Yield: 86%; m-p. 187–190 °C; FT-IR (solid, cm^−1^): 3318, 1694, 1664, and 1580, 1362, 1260, 1160, and 1092; ^1^H-NMR (CDCl_3_): δ 8.54 (1H, s, NH), 7.30 (1H, C-NH), 4.88 (2H, NH-NH_2_), 7.11–7.28 (5H, m), 4.97 (CH-NH), 2.93–3.14 (2H, CH_2_), 1.36 (9H, s); ^13^C-NMR (CDCl_3_): δ 28.3, 38.5, 55.2, 79.7, 126.0, 126.6, 126.7, 128.0, 128.5, 155.3, 143.2, 155.9, 177.3; HRMS (ESI+) *m*/*z*: 302.1477 [M + Na]^+^.

In the fourth step, a solution of aldehyde (0.6 mmol) added to a stirred solution of **1** (0.5 mmol) in EtOH (5 mL) at room temperature for 24 h. The progress of the reaction was monitored with TLC system of EtOAc/*n*-hexane (3:7). After completion of the reaction, the product (**3**) was filtered, washed with *n*-hexane for removing any excess of aldehyde. The resulting residue was redissolved in DMSO (2 mL), followed by addition of potassium carbonate (3 mmol) and iodine (1.2 mmol) in sequence. The reaction mixture was stirred at 100 °C until the conversion was complete (1−4 h, monitored by TLC,). After being cooled to room temperature, it was treated with 5% Na_2_S_2_O_3_ (20 mL), extracted with EtOAc (10 mL, three times). The combined organic layer was washed with brine (10 mL × 1), dried over anhydrous sodium sulfate, and concentrated. The given residue was purified through preparative thin layer chromatography (TLC) using a mobile phase of EtOAc and *n*-hexane (3:7) to afford the desired oxadiazoles. All compounds were obtained in fare yields ranging from 57 to 64%. The structures of all compounds were established by HRMS, ^1^H- and ^13^C-NMR and same procedure was use for the synthesis of other compounds.

#### 3.2.1. Tert-Butyl (S)-(1-phenyl-2-(5-(pyridin-2-yl)-1,3,4-oxadiazol-2-yl) ethyl) carbamate (**4a**)

White solid powder; Yield: 57%; FTIR (solid, cm^−1^): 3320, 1690, 1666, and 1585, 1365, 1264, 1160, and 1094; ^1^H-NMR (CDCl_3_): δ 8.76 (1H), 8.18 (1H), 7.86 (1H), 7.45 (1H), 7.25–7.10 (5H, m), 5.40 (1H, NH), 5.19 (1H, CH), 3.22, 3.35 (2H), 1.37 (9H, s); ^13^C-NMR (CDCl_3_): δ 28.2, 39.9, 48.5, 80.4, 123.2, 126.0, 127.2, 128.7, 129.4, 135.2, 137.3, 143.3, 150.3, 164.2, 167.1; HRMS (ESI^+^) *m*/*z*: 366.1756 [M + H]^+^.

#### 3.2.2. Tert-Butyl (S)-(2-(5-(2-hydroxyphenyl)-1,3,4-oxadiazol-2-yl)-1-phenylethyl) carbamate (**4b**)

Solid powder; Yield: 56%; FTIR (solid, cm^−1^): 3318, 1689, 1670, and 1594, 1445, 1375, 1340, 1302 and 1160; ^1^H-NMR (CDCl_3_): δ 10.86 (1H, OH, s), 8.15 (1H), 7.65 (1H), 7.42 (1H), 7.30 (1H), 7.25–6.72 (5H, m), 5.36 (1H, NH), 5.09 (1H, CH), 3.29, 3.28 (2H), 1.41 (9H, s); ^13^C-NMR (CDCl_3_): δ 28.2, 39.8, 48.6, 81.2, 86.8, 109.5, 119.9, 121.5, 124.9, 126.8, 127.5, 128.7, 128.9, 129.3, 133.8, 134.7, 143.3, 154.8, 157.7, 165.8; HRMS (ESI^+^) *m*/*z*: 382.1814 [M + H]^+^.

#### 3.2.3. Tert-Butyl(S)-(1-phenyl-2-(5-phenyl-1,3,4-oxadiazol-2-yl)ethyl) carbamate (**4c**)

Pale yellow Color; Yield: 58%; FTIR (solid, cm^−1^): 3336, 1710, 1668, 1608, 1375, 1335, 1290 and 1080; ^1^H-NMR (CDCl_3_, 600 MHz): δ 7.88–7.39 (5H, m), 7.22–7.07 (5H, m), 5.47 (1H, NH), 5.25 (1H, CH), 3.23, 3.20 (2H), 1.33 (9H, s); ^13^C-NMR (150 MHz, CDCl_3_): δ 28.2, 29.6, 48.5, 80.3, 123.6, 126.8, 127.1, 128.6, 129.0, 132.9, 131.7, 135.6, 154.9, 164.9, 166.2; HRMS (ESI^+^) *m*/*z*: 366.1948 [M + H]^+^.

#### 3.2.4. Tert-Butyl(S)-(2-(5-(4-methoxyphenyl)-1,3,4-oxadiazol-2-yl)-1-phenylethyl) carbamate (**4d**)

White solid powder; Yield: 57%; FTIR (solid, cm^−1^): 3324, 1704, 1672, 1602, 1452, 1370, 1268, 1160, and 1020; ^1^H-NMR (CDCl_3_): δ 7.81 (1H), 7.80 (1H), 6.90 (1H), 6.89 (1H), 7.24–7.07 (5H, m), 5.47 (1H, NH), 5.22 (1H, CH), 3.78 (3H, CH_3_), 3.23,3.17 (2H), 1.32 (9H, s); ^13^C-NMR (CDCl_3_): δ 28.2, 29.6, 48.5, 55.4, 114.4, 116.0, 127.1, 128.6, 129.3, 135.6, 154.9, 162.3, 164.8, 165.6; HRMS (ESI^+^) *m*/*z*: 396.1960 [M + H]^+^.

#### 3.2.5. Tert-Butyl(S)-(2-(5-(4-hydroxy-3-methoxyphenyl)-1,3,4-oxadiazol-2-yl)-1-phenylethyl) carbamate (**4e**)

Yellow powder; Yield: 56%; FTIR (solid, cm^−1^): 3333, 1696, 1668, 1590, 1450, 1370, 1252, 1160, 1030; ^1^H-NMR (CDCl_3_): δ 9.80 (1H, OH, s), 7.63 (1H), 7.51 (1H), 7.25–7.11 (5H, m), 6.87 (1H), 5.43 (1H, NH), 5.31 (1H, CH), 4.44 (3H, OCH_3_), 3.94,3.86 (2H), 1.38 (9H, s); ^13^C-NMR (CDCl_3_): δ 28.2, 34.4, 38.5, 48.6, 40.9, 52.5, 56.1, 107.7, 109.5, 119.9, 121.5, 124.9, 126.8, 128.7, 129.3, 136.5, 145.0, 155.3, 167.8, 190.8; HRMS (ESI^+^) *m*/*z*: 436.2279 [M + H]^+^.

#### 3.2.6. Tert-Butyl(S)-(2-(5-(2-hydroxy-3-methoxyphenyl)-1,3,4-oxadiazol-2-yl)-1-phenylethyl) carbamate (**4f**)

Crystalline powder; Yield: 61%; FTIR (solid, cm^−1^): 3320, 1690, 1664, 1594, 1450, 1405, 1358, 1257, 1164, and 1090; ^1^H-NMR (CDCl_3_, 600 MHz): δ 9.90 (1H, OH, s), 8.17 (1H), 7.93 (1H), 7.29–7.10 (5H, m), 6.85 (1H), 5.27 (1H, NH), 4.45 (1H, CH), 3.91 (3H, OCH3), 3.91,3.87 (2H), 1.37 (9H, s); 13C-NMR (150 MHz, CDCl_3_): δ 28.2, 34.4, 38.1, 56.3, 113.7, 114.2, 117.5, 119.5, 121.4, 124.5, 126.9, 128.4, 129.5, 136.3, 145.6, 151.1, 155.9, 167.4, 172.8, 196.6; HRMS (ESI^+^) *m*/*z*: 436.1883 [M + H]^+^.

#### 3.2.7. Tert-Butyl(S)-(2-(5-(2-nitrophenyl)-1,3,4-oxadiazol-2-yl)-1-phenylethyl) carbamate (**4g**)

Yellow powder; Yield: 56%; FTIR (solid, cm^−1^): 3310, 1703, 1666, 1614, 1455, 1346, 1324, 1236, 1059; ^1^H-NMR (CDCl_3_): δ 8.05 (1H), 7.87 (1H), 7.67 (1H), 7.71 (1H), 7.29–7.13 (5H, m), 5.34 (1H, NH), 5.13 (1H, CH), 3.30,3.18 (2H), 1.38 (9H, s); ^13^C-NMR (CDCl_3_): δ 28.2, 39.8, 48.4, 118.7, 124.8, 127.3, 128.7, 129.4, 131.9, 132.6, 133.3, 135.0, 148.1, 154.8, 161.8, 167.3; HRMS (ESI^+^) *m*/*z*: 411.1694 [M + H]^+^.

#### 3.2.8. Tert-Butyl(S)-(2-(5-(4-nitrophenyl)-1,3,4-oxadiazol-2-yl)-1-phenylethyl) carbamate (**4h**)

Light yellow powder; Yield: 61%; FTIR (solid, cm^−1^): 3322, 1701, 1664, 1610, 1430, 1365, 1345, 1208, and 1163; ^1^H-NMR (CDCl_3_): δ 8.38 (1H), 8.37 (1H), 8.17 (1H), 8.16 (1H), 7.32–7.17 (5H, m), 5.41 (1H, NH), 5.20 (1H, CH), 3.34,3.33 (2H), 1.45 (9H, s); ^13^C-NMR (CDCl_3_): δ 28.2, 29.7, 40.0, 48.6, 124.3, 124.5, 126.9, 127.4, 127.8, 128.8, 129.0, 129.1, 129.3, 135.1, 149.7, 154.8, 163.2, 167.4; HRMS (ESI^+^) *m*/*z*: 411.1705 [M + H]^+^.

#### 3.2.9. Tert-Butyl (S)-(2-(5-(4-hydroxyphenyl)-1,3,4-oxadiazol-2-yl)-1-phenylethyl) carbamate (**4i**)

White solid powder; Yield: 59%; FTIR (solid, cm^−1^): 3330, 1705, 1668, and 1608; ^1^H-NMR (CDCl_3_): δ 7.78 (1H), 7.77 (1H), 7.26 (1H), 7.20 (1H), 7.12–6.86 (5H, m), 5.30 (1H, NH), 5.16 (1H, CH), 3.29,3.24 (2H), 1.38 (9H, s); ^13^C-NMR (CDCl_3_): δ 28.2, 29.3, 29.7, 29.7, 31.9, 40.0, 48.5, 80.7, 116.0, 116.1, 127.2, 128.7, 128.9, 129.4, 135.4, 158.9, 164.9, 165.5; HRMS (ESI^+^) *m*/*z*: 382.1463 [M + H]^+^.

#### 3.2.10. Tert-Butyl (S)-(2-(5-(3,4-dimethoxyphenyl)-1,3,4-oxadiazol-2-yl)-1-phenylethyl) carbamate (**4j**)

White amorphous powder; Yield: 64%; FTIR (solid, cm^−1^): 3326, 1701, 1670, 1595, 1420, 1370, 1336, 1252, 1208, and 1143; ^1^H-NMR (CDCl_3_): δ 7.50 (1H), 7.49 (1H), 7.45 (1H), 7.27–7.12 (5H, m), 6.90 (1H), 5.32 (1H, NH), 5.18 (1H, CH), 3.92 (6H, OCH3), 3.27,3.26 (2H), 1.40 (9H, s); ^13^C-NMR (CDCl3): δ 28.3, 40.1, 48.5, 56.0, 56.1, 80.5, 109.5, 111.1, 116.2, 120.4, 127.2, 128.6, 129.4, 135.5, 149.3, 152.1, 164.9, 165.7; HRMS (ESI^+^) *m*/*z*: 426.2066 [M + H]^+^.

#### 3.2.11. Tert-Butyl (S)-(2-(5-(2,5-dimethoxyphenyl)-1,3,4-oxadiazol-2-yl)-1-phenylethyl) carbamate (**4k**)

Colorless solid; Yield: 61%; FTIR (solid, cm^−1^): 3328, 1711, 1667, 1602, 1524, 1342, 1272, 1228, 1155, and 1066; ^1^H-NMR (CDCl_3_): δ 7.30 (1H), 7.24–7.11 (5H, m), 7.03 (1H), 6.96 (1H), 5.36 (1H, NH), 5.22 (1H, CH), 3.86,3.78 (6H, OCH_3_), 3.30,3.27 (2H), 1.40 (9H, s); ^13^C-NMR (CDCl_3_): δ 28.3, 40.0, 48.5, 56.0, 56.6, 60.4, 80.3, 113.1, 113.7, 114.5, 119.4, 127.1, 128.6, 129.4, 135.5, 152.3, 153.4, 163.6, 165.8; HRMS (ESI^+^) *m*/*z*: 426.2065 [M + H]^+^.

#### 3.2.12. Tert-Butyl (S)-(2-(5-(4-chlorophenyl)-1,3,4-oxadiazol-2-yl)-1-phenylethyl) carbamate (**4l**) 

White powder; Yield: 57%; FTIR (solid, cm^−1^): 3350, 1698, 1663, and 1603, 1532, 1336, 1266, 1224, 1172, and 1066; ^1^H-NMR (CDCl_3_): δ 7.88 (1H), 7.87 (1H), 7.45 (1H), 7.44 (1H), 7.27–7.12 (5H, m), 5.32 (1H, NH), 5.16 (1H, CH), 3.28,3.27 (2H), 1.40 (9H, s); ^13^C-NMR (CDCl_3_): δ 28.2, 29.7, 39.9, 48.6, 80.6, 122.2, 127.3, 128.2, 128.7, 129.3, 129.4, 135.3, 138.1, 154.8; HRMS (ESI^+^) *m*/*z*: 400.1467 [M + H]^+^.

### 3.3. Carbonic Anhydrase II Inhibition Assay

A total reaction volume of 200 µL containing 20 µL of the synthetic compounds **4a**–**l** prepared in DMSO, followed by the addition of 140 µL of the HEPES-tris buffer, 20 µL of purified bovine erythrocyte CA-II (0.15 mg/mL) prepared in buffer, and 20 µL of a solution of 4-nitrophenyl acetate [36,44]. 20 µL of tested compounds were incubated with the enzyme carbonic anhydrase II (EC 4.2.1.1) for 15 min in 96-well flat bottom plate. The rate of product formation was monitored with the addition of 20 µL of 4-NPA as substrate, prepared in ethanol at the final concentration of 0.7 mM at 25 °C for 30 min with regular intervals of 1 min, by using spectrophotometer (xMark^TM^ Microplate, Bio-Rad, Hercules, CA, USA). HEPES-tris was used as a buffer for the reaction at the final concentration of 20 mM at pH 7.4.

### 3.4. Molecular Docking

Docking was conducted on Molecular Operating Environment [45] using X-ray crystal structure of human carbonic anhydrase II complexed with acetazolamide (PDB code: 3HS4, resolution: 1.10 Å) [62]. The docking performance of MOE was tested previously [36,44] by re-docking experiment which shows that MOE is efficient in docking of CA-II inhibitors. In the re-docking, the X-ray conformation of acetazolamide was re-docked at its cognate binding site, where it was docked with RMSD = 0.61 Å. The re-docked conformation is shown in Figure S37. The protein file was prepared for docking by QuickPrep module of MOE which adds missing hydrogen atoms on residues and calculate partial charges using pre-defined force field (we applied Amber10:EHT force field). The two-dimensional (2D-) structures of ligands were prepared by ChemDraw, later converted into 3D-form by MOE using WASH module of MOE which adds hydrogen atoms and partial charges on ligands. Subsequently, the 3D-structure of each compound was minimized until the gradient was reached to 0.1RMS kcal/mol/Å. After the preparation of protein and ligand files, docking was performed by Triangle Matcher docking algorithm and London dG scoring function. Thirty docked conformation of each ligand was saved, and the best docked orientation was selected based on the docking score and binding interactions. The pharmacokinetic profile of the active hits was predicted through SwissADME server using BOILED-Egg model [61,63].

## 4. Conclusions

In summary, a series of twelve new 3-phenyl-β-alanine 1,3,4-oxadiazoles **4a**–**l** have been synthesized and CA-II were performed in vitro. From all derivatives, compounds **4a** (12.1 µM) and **4c** (13.8 µM) exhibited the most potent activity against CA-II enzyme. In addition, structure-activity relationship of the active compounds has been established. When the results were compared, it was observed that these molecules show more effective inhibition than the standard inhibitor. Based on molecular docking strategy, we observed the mode of binding interaction of the active hits with the active site residues of CA-II, which suggests that the compounds mainly interact with the Thr199 and Thr200, thus inhibit the activity of CA-II. Based on these results, we suggest that these molecules can be used against CA-II related diseases.

## Figures and Tables

**Table 1:** Carbonic anhydrase-II activity of the compounds **4a**–**l**.

Compounds	R	% Inhibition	IC_50_ ± SEM (µM)
**4a**	**2a** 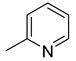	96.2	12.1 ± 0.86
**4b**	**2b** 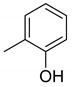	93.5	19.1 ± 0.88
**4c**	**2c** 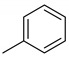	95.5	13.8 ± 0.64
**4d**	**2d** 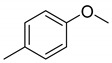	29.0	N/A
**4e**	**2e** 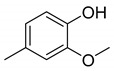	97.3	26.6 ± 0.80
**4f**	**2f** 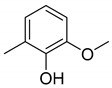	93.8	25.1 ± 1.04
**4g**	**2g** 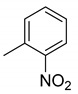	79.5	21.5 ± 0.99
**4h**	**2h** 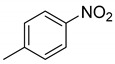	94.6	20.7 ± 1.13
**4i**	**2i** 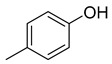	58.0	18.1 ± 1.31
**4j**	**2j** 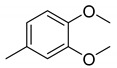	96.7	26.6 ± 1.47
**4k**	**2k** 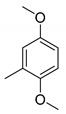	87.8	22.4 ± 1.43
**4l**	**2l** 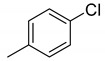	67.6	53.6 ± 0.96
Standard	Acetazolamide	84.6	18.2 ± 1.43

N/A = Not active, SEM = Standard error mean.

**Table 2:** Molecular docking results of active inhibitors.

Compounds	Score (kcal/mol)	Interactions			
		Ligand Atom	Receptor Atom	Bond Type	Distance (Å)
**4a**	−8.96	O25	N-THR199	HBA	1.87
		O25	N-THR200	HBA	2.37
		6-ring	HOH-1247	π-H	3.53
**4c**	−8.2	N22	OG1-THR200	HBD	1.97
		O25	N-THR200	HBA	2.39
**4i**	−8.05	N22	OG1-THR200	HBD	2.95
		O25	N-THR200	HBA	2.61
**4b**	−7.88	O25	N-THR200	HBA	2.26
		O25	OG1-THR 200	HBA	1.80
		6-ring	O-HOH1247	π-H	3.46
**4h**	−7.63	O25	OG1-THR200	HBA	2.23
**4g**	−7.68	O26	N-THR199	HBA	1.87
**4k**	−7.80	O25	OG1-THR200	HBA	2.11
**4f**	−7.10	O21	NE2-GLN92	HBA	2.97
		O25	N-THR200	HBA	2.21
**4e**	−7.09	N22	O-HOH1191	HBD	1.99
		6-ring	6-ring-PHE131	π-π	3.01
**4j**	−7.10	O25	N-THR199	HBA	2.20
**4l**	−6.23	N22	OG1-THR200	HBD	1.82
		O25	N-THR200	HBA	2.08

HBA = Hydrogen bond acceptor, HBD = Hydrogen bond donor.

**Table 3:** The pharmacokinetic profile of active inhibitors.

Molecule	NRB	HBA	HBD	TPSA	iLOGP	GIA	BBBP	PgpS	Lipinski V	PAINS Alerts
**4a**	8	6	1	90.14	3	High	No	Yes	0	0
**4b**	8	6	2	97.48	3.34	High	No	No	0	0
**4c**	8	5	1	77.25	3.54	High	No	No	0	0
**4e**	9	7	2	106.71	3.56	High	No	No	0	0
**4f**	9	7	2	106.71	3.45	High	No	No	0	0
**4g**	9	7	1	123.07	2.96	High	No	No	0	0
**4h**	9	7	1	123.07	3.11	High	No	No	0	0
**4i**	8	6	2	97.48	3.15	High	No	No	0	0
**4j**	10	7	1	95.71	3.81	High	No	No	0	0
**4k**	10	7	1	95.71	3.64	High	No	No	0	0
**4l**	8	5	1	77.25	3.75	High	No	No	0	0
AZM	3	6	1	178.33	0	Low	No	No	0	0

NRB = Number of rotatable bonds, HBA = H-bond acceptors, HBD = H-bond donors, TPSA = Topological polar surface area, GIA = Gastrointestinal absorption, BBBP = Blood brain barrier permeant, PgpS = P glycoprotein substrate, Lipinski V = Violation of lipinski rule of 5.

## Data Availability

The spectroscopic data is available in the supporting information to the researchers.

## Supplementary Materials

The following supporting information can be downloaded, Figures S1–S36: ^1^H- (CDCl_3_, 600 MHz), ^13^C-NMR and HRESIMS of the compounds **4a**–**l**, Figure S37: The re-docked orientation of acetazolamide.

## Sample Availability

The data presented in this study is available in the Supplementary Material.
