# Supplementary material for: Design, Synthesis and Molecular Docking Study of Novel 3-Phenyl-β-Alanine-Based Oxadiazole Analogues as Potent Carbonic Anhydrase II Inhibitors
Source: Molecules. 2022 Jan 26;27(3):816. doi: 10.3390/molecules27030816 (PMC8838037; doi:10.3390/molecules27030816)
Supplement: Supplementary file 1 [file molecules-27-00816-s001.zip › molecules-1549767-supplementary.pdf]

molecules

MDPI

Suporting Information

Design, Synthesis and Molecular Docking Study of Novel Phenylalanine Based Oxadiazole Analogues as Potent Carbonic Anhydrase II Inhibitors

Kashif Rafiq [1,2], Najeeb Ur Rehman [1,*], Sobia Ahsan Halim[1], Majid Khan [1,3], Ajmal Khan [1] and Ahmed Al-Harrasi [1,*]

[1] Natural and Medical Sciences Research Center, University of Nizwa, P.O Box 33, Birkat Al Mauz, Nizwa 616, Oman; kashifrafiq@unizwa.edu.om (K.R.); sobia_halim@unizwa.edu.om (S.A.H.); majidk166@yahoo.com (M.K.); ajmalkhan@unizwa.edu.om (A.K.)
[2] Department of Chemistry, Abdul Wali Khan University Mardan, Mardan 23200, Pakistan
[3] H. E. J. Research Institute of Chemistry, International Center for Chemical and Biological Sciences, University of Karachi, Karachi 75270, Pakistan
* Correspondence: najeeb@unizwa.edu.om (N.U.R.); aharrasi@unizwa.edu.om (A.A.-H.); Tel.: +968-2544-6328 (A.A.-H.); Fax: +968-2544-6612 (A.A.-H.)

List of Figures

Figure S1. ^{1}H-NMR (CDCl3, 600 MHz) of compound **4a**
Figure S2. ^{13}C NMR (CDCl3, 125 MHz) of compound **4a**
Figure S3: HRMS (ESI^{+}) of compound **4a**
Figure S4. ^{1}H-NMR (CDCl3, 600 MHz) of compound **4b**
Figure S5. ^{13}C NMR (CDCl3, 125 MHz) of compound **4b**
Figure S6: HRMS (ESI^{+}) of compound **4b**
Figure S7. ^{1}H-NMR (CDCl3, 600 MHz) of compound **4c**
Figure S8. ^{13}C NMR (CDCl3, 125 MHz) of compound **4c**
Figure S9: HRMS (ESI^{+}) of compound **4c**
Figure S10. ^{1}H-NMR (CDCl3, 600 MHz) of compound **4d**
Figure S11. ^{13}C NMR (CDCl3, 125 MHz) of compound **4d**
Figure S12: HRMS (ESI^{+}) of compound **4d**
Figure S13. ^{1}H-NMR (CDCl3, 600 MHz) of compound **4e**
Figure S14. ^{13}C NMR (CDCl3, 125 MHz) of compound **4e**
Figure S15: HRMS (ESI^{+}) of compound **4e**
Figure S16. ^{1}H-NMR (CDCl3, 600 MHz) of compound **4f**
Figure S17. ^{13}C NMR ($CDCl_3$, 125 MHz) of compound **4f**
Figure S18: HRMS (ESI^{+}) of compound **4f**
Figure S19. ^{1}H-NMR ($CDCl_3$, 600 MHz) of compound **4g**
Figure S20. ^{13}C NMR (CDCl3, 125 MHz) of compound **4g**
Figure S21: HRMS (ESI^{+}) of compound **4g**
Figure S22. ^{1}H-NMR (CDCl3, 600 MHz) of compound **4h**

Figure S23. ^{13}C NMR (CDCl3, 125 MHz) of compound **4h**

Figure S24: HRMS (ESI+) of compound **4h**

Figure S25. ^{1}H-NMR (CDCl3, 600 MHz) of compound **4i**

Figure S26. ^{13}C NMR (CDCl3, 125 MHz) of compound **4i**

Figure S27: HRMS (ESI+) of compound **4i**

Figure S28. ^{1}H-NMR (CDCl3, 600 MHz) of compound **4j**

Figure S29. ^{13}C NMR (CDCl3, 125 MHz) of compound **4j**

Figure S30: HRMS (ESI+) of compound **4j**

Figure S31. ^{1}H-NMR (CDCl3, 600 MHz) of compound **4k**

Figure S32. ^{13}C NMR (CDCl3, 125 MHz) of compound **4k**

Figure S33: HRMS (ESI+) of compound **4k**

Figure S34. ^{1}H-NMR (CDCl3, 600 MHz) of compound **4l**

Figure S35. ^{13}C NMR (CDCl3, 125 MHz) of compound **4l**

Figure S36: HRMS (ESI+) of compound **4l**

Figure S37: The re-docked orientation of acetazolamide.

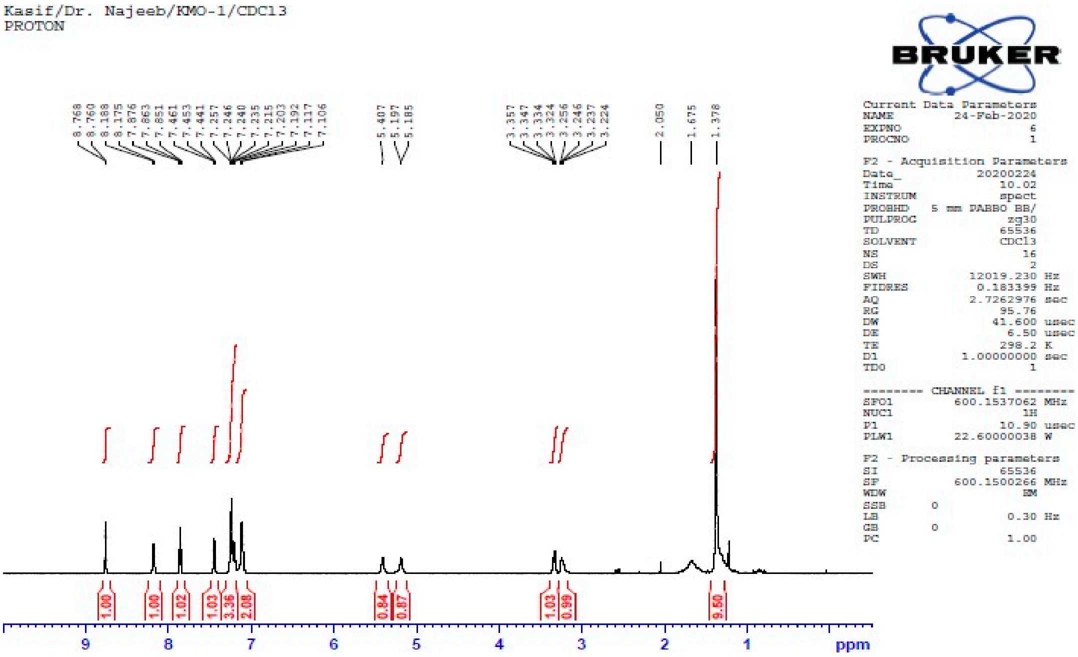

Figure S1. ^{1}H-NMR (CDCl$_3$, 600 MHz) of compound 4a fix font and size of figure labels.

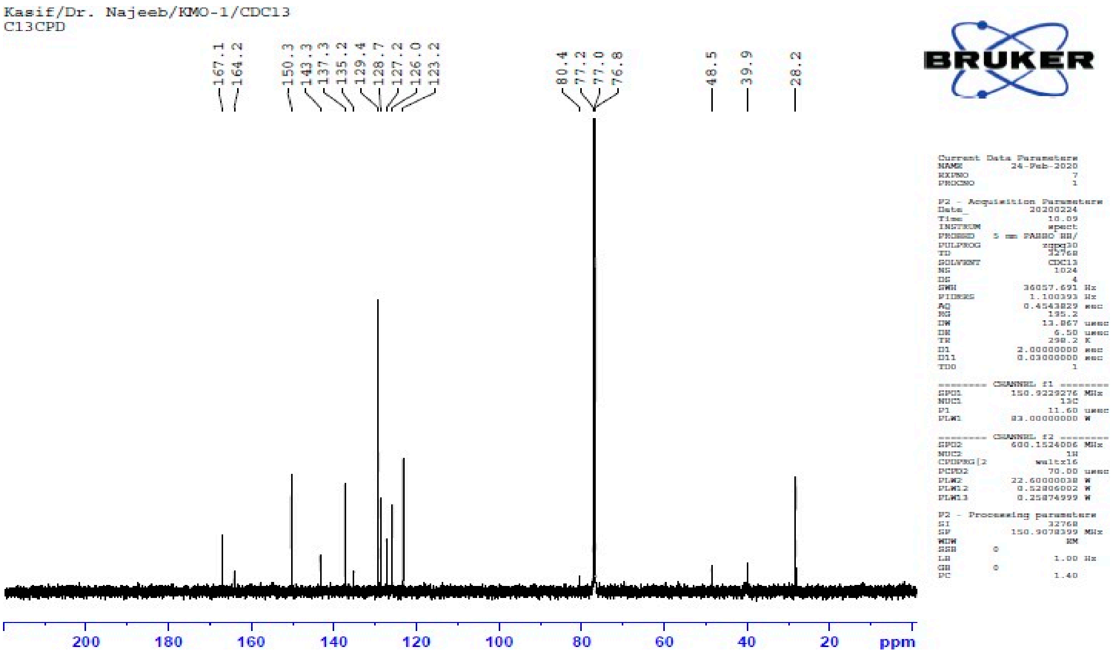

Figure S2. ^{13}C-NMR (CDCl3, 600 MHz) of compound **4a.**

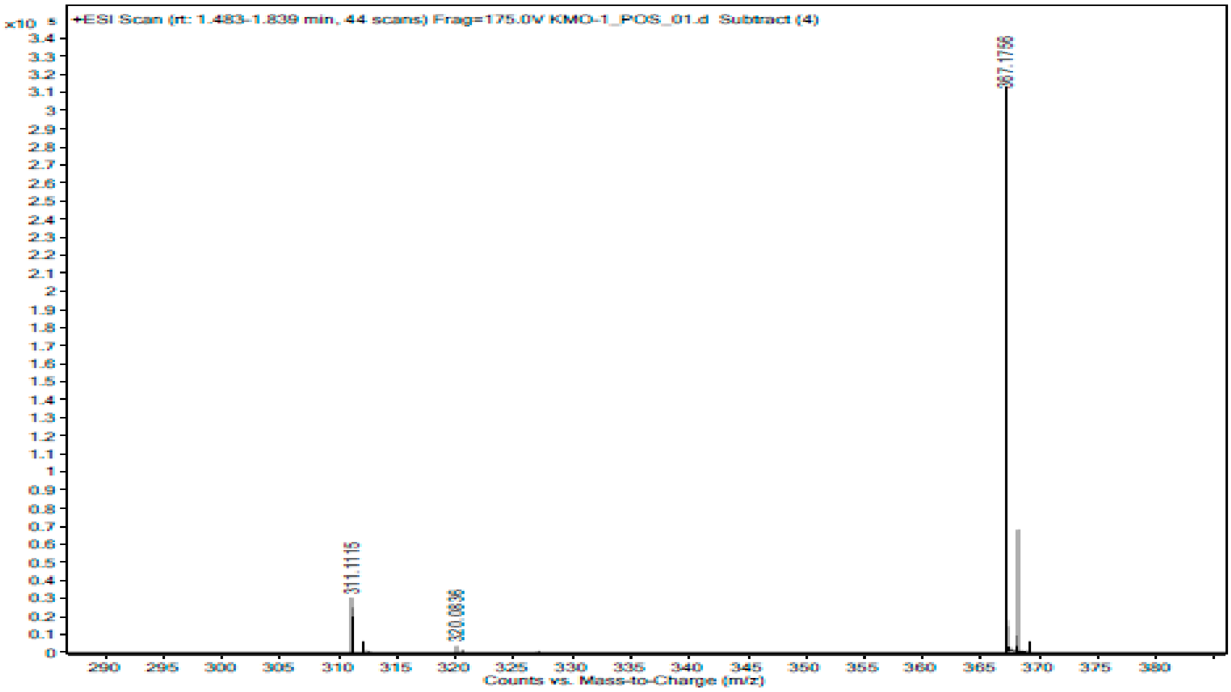

Figure S3. HRMS (ESI$^+$) of compound **4a.**

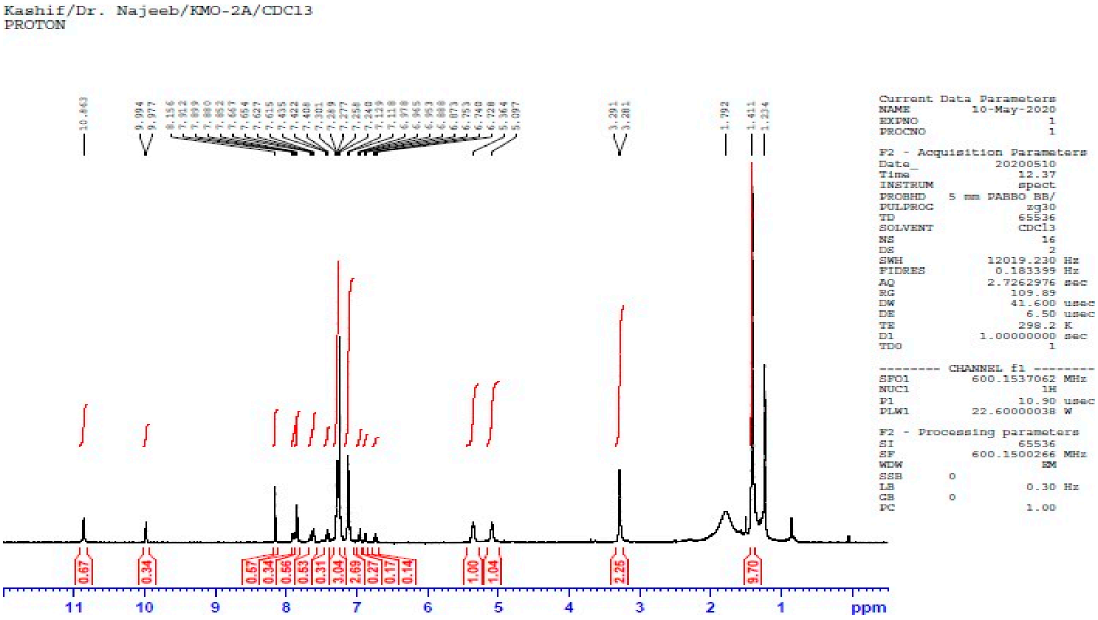

Figure S5. ^{13}C-NMR (CDCl3, 125 MHz) of compound **4b.**

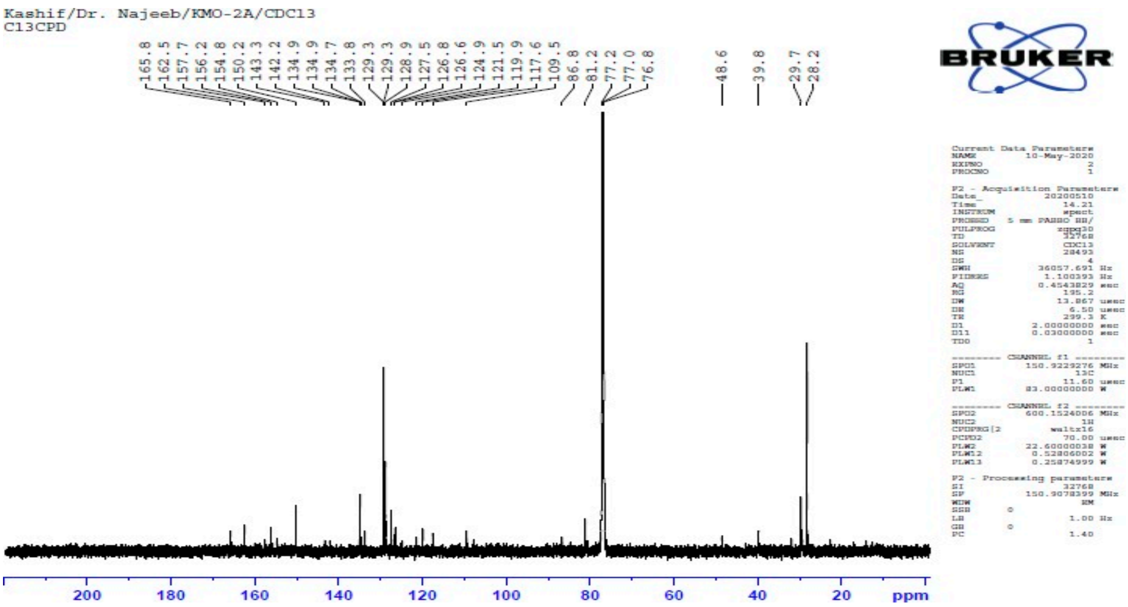

Figure S4. ^{1}H-NMR (CDCl3, 600 MHz) of compound **4b.**

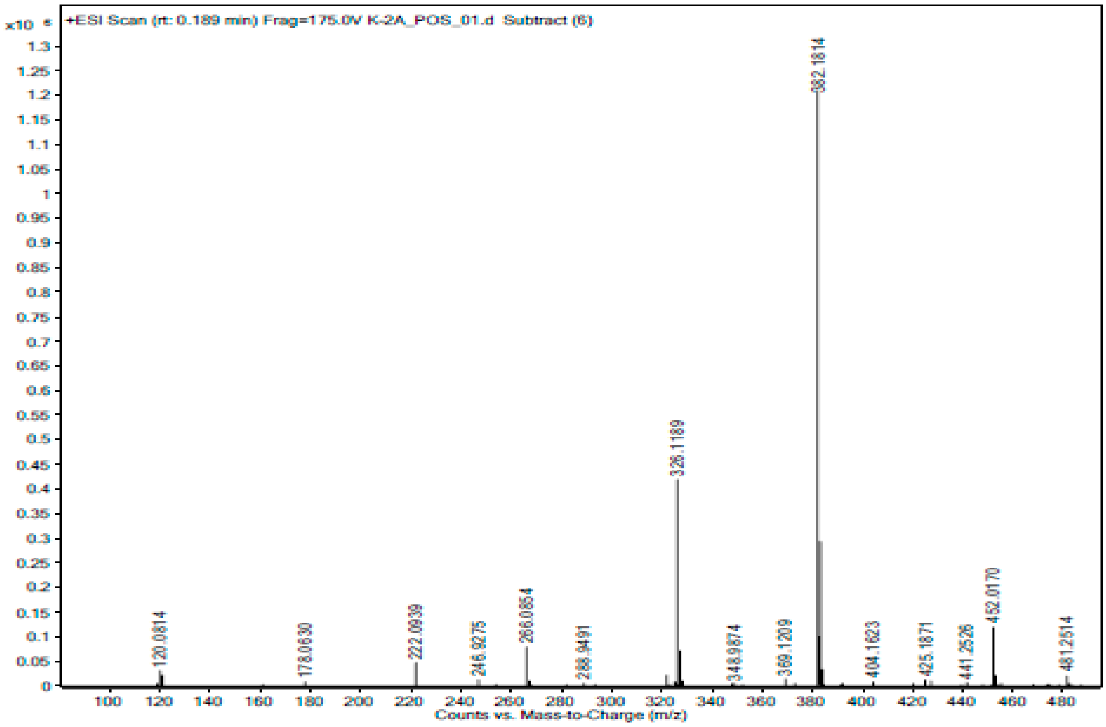

Figure S6. HRMS (ESI+) of compound **4b.**

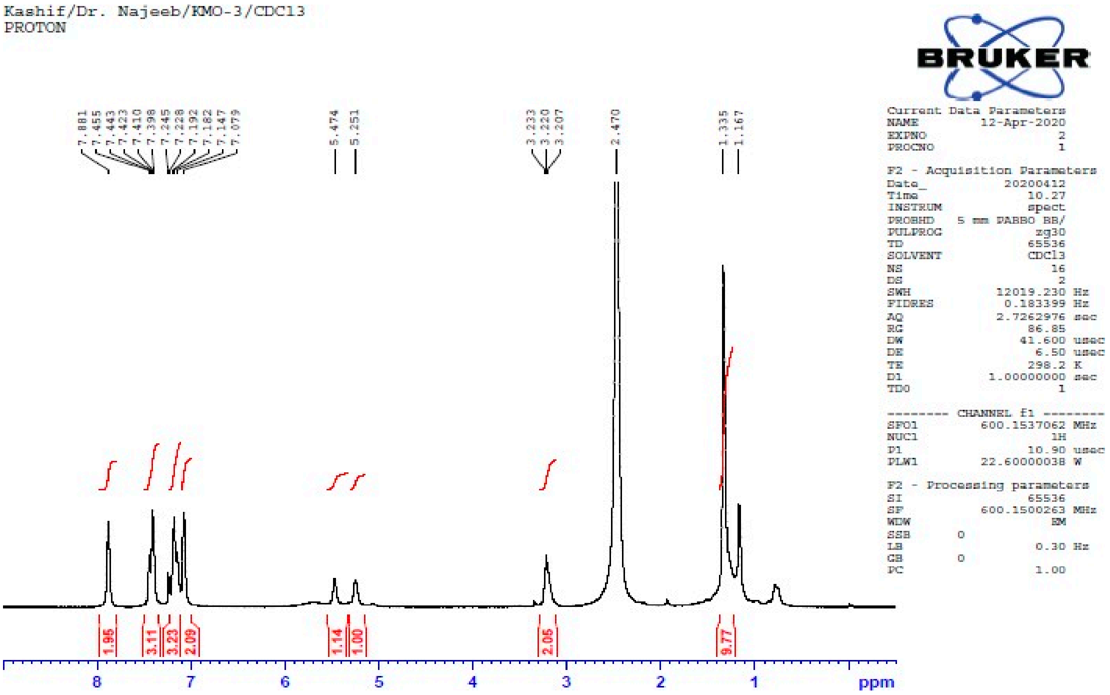

Figure S7. 1H-NMR (CDCl3, 600 MHz) of compound **4c.**

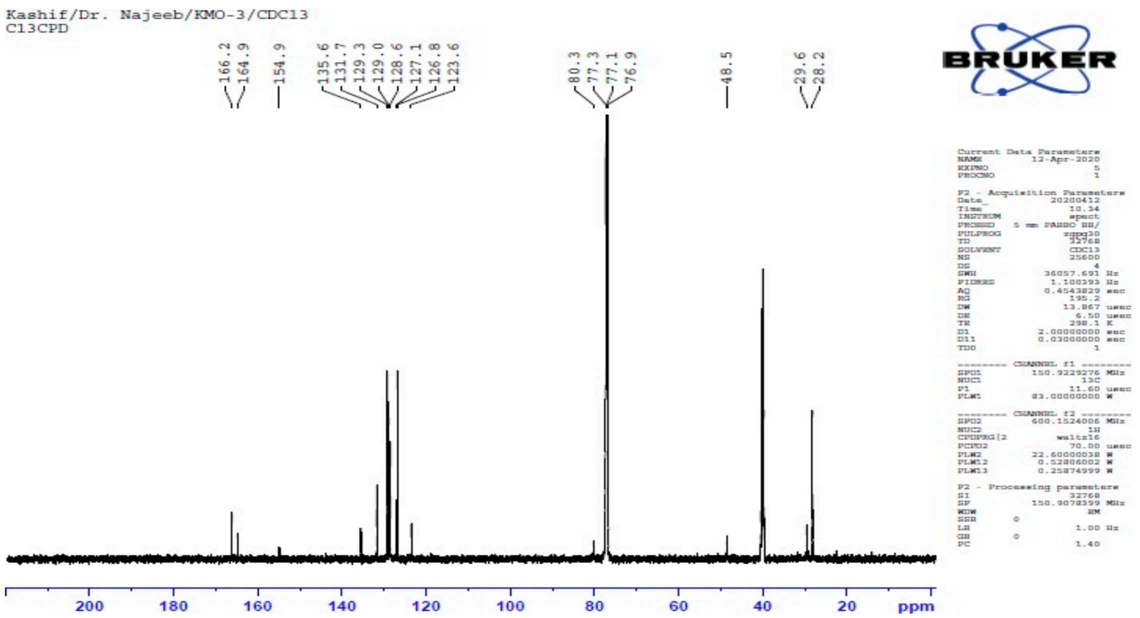

Figure S8. ^{13}C-NMR ($CDCl_3$, 125 MHz) of compound **4c.**

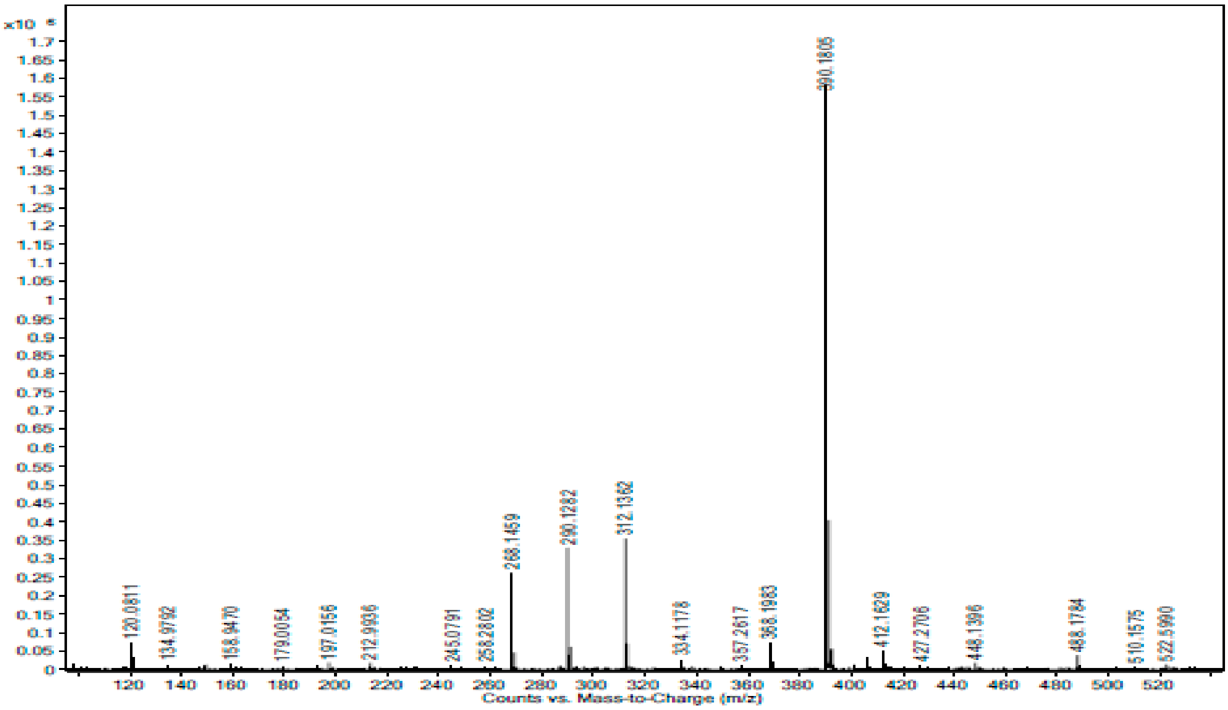

Figure S9. HRMS (ESI^+) of compound **4c.**

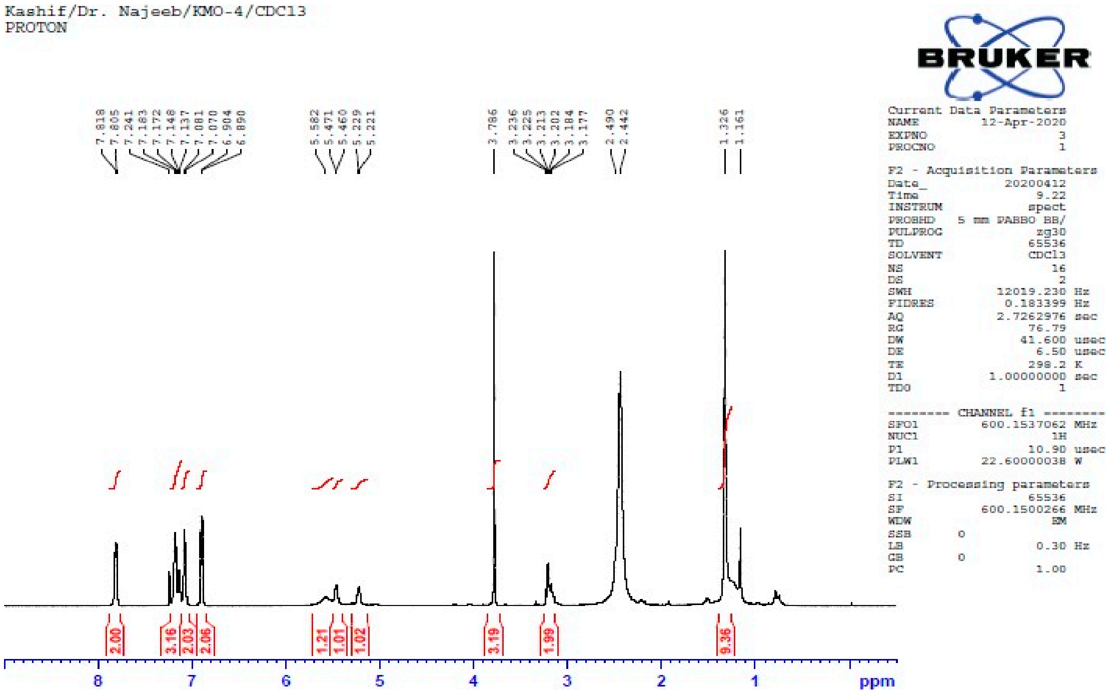

Figure S10. ^{1}H-NMR ($CDCl_3$, 600 MHz) of compound **4d.**

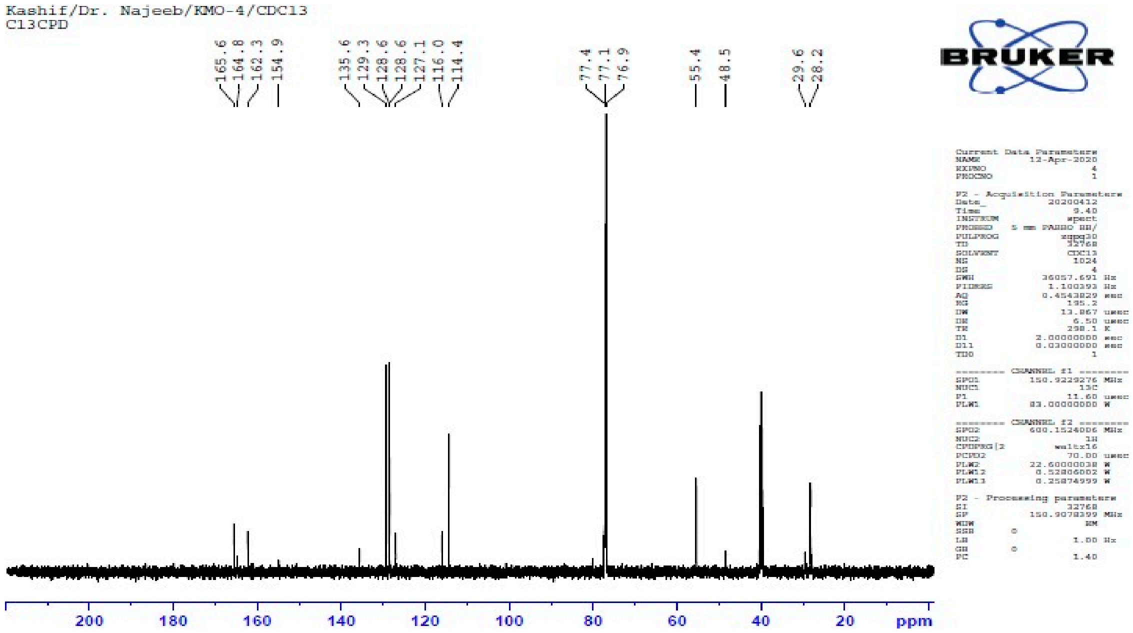

Figure S11. ^{13}C-NMR ($CDCl_3$, 125 MHz) of compound **4d.**

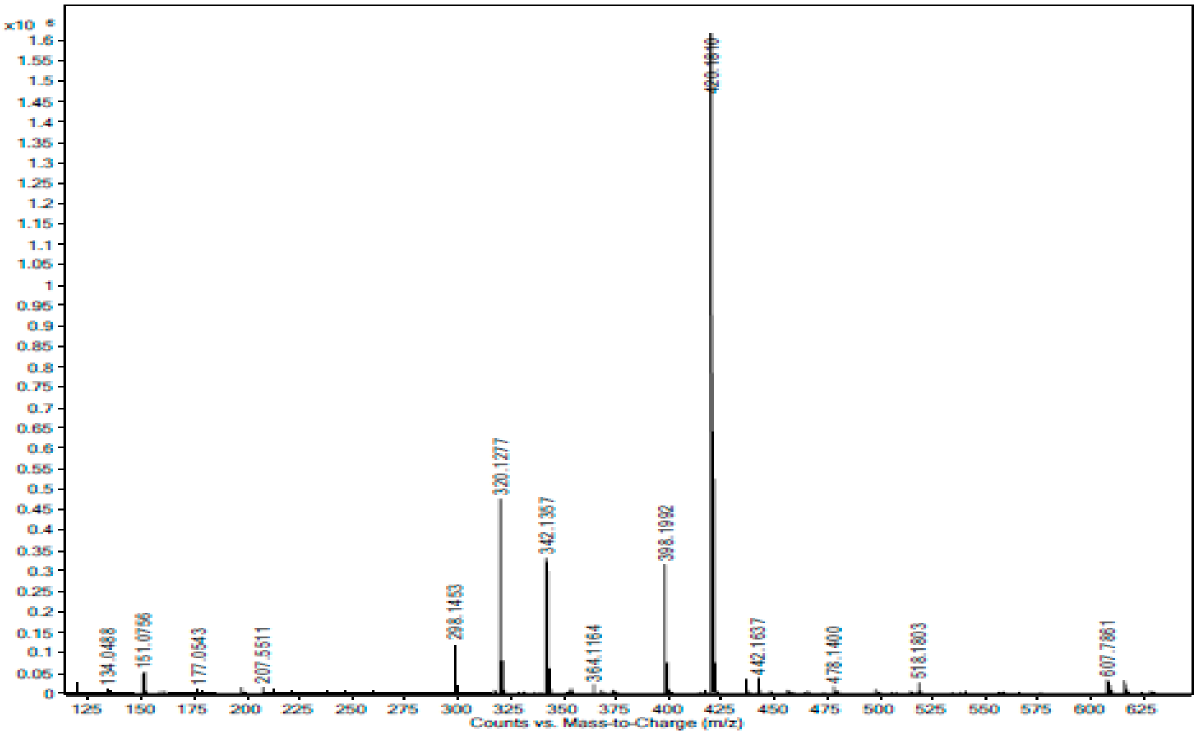

Figure S12. HRMS (ESI+) of compound **4d.**

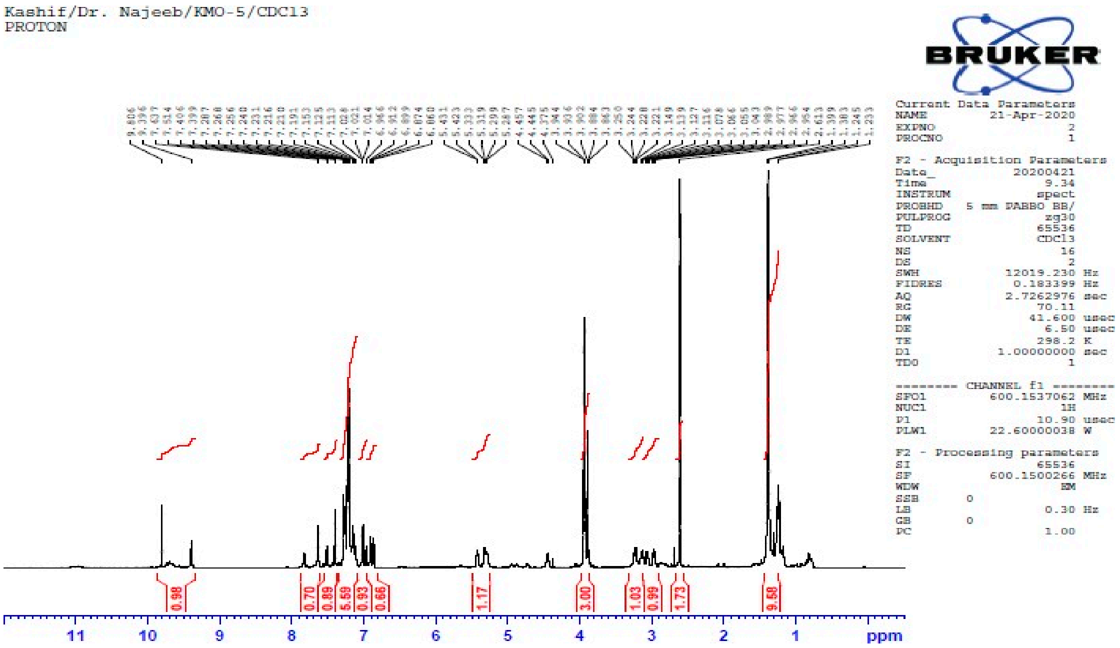

Figure S13. 1H-NMR (CDCl3, 600 MHz) of compound **4e.**

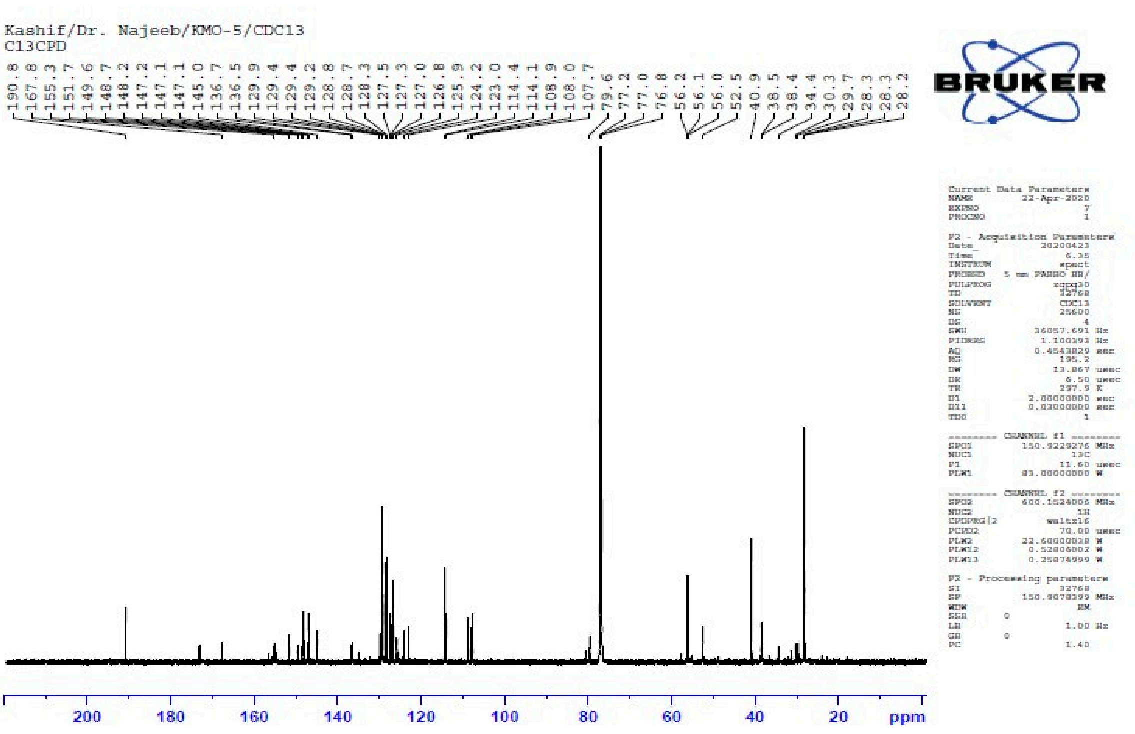

Figure S14. ^{13}C-NMR (CDCl3, 125 MHz) of compound **4e.**

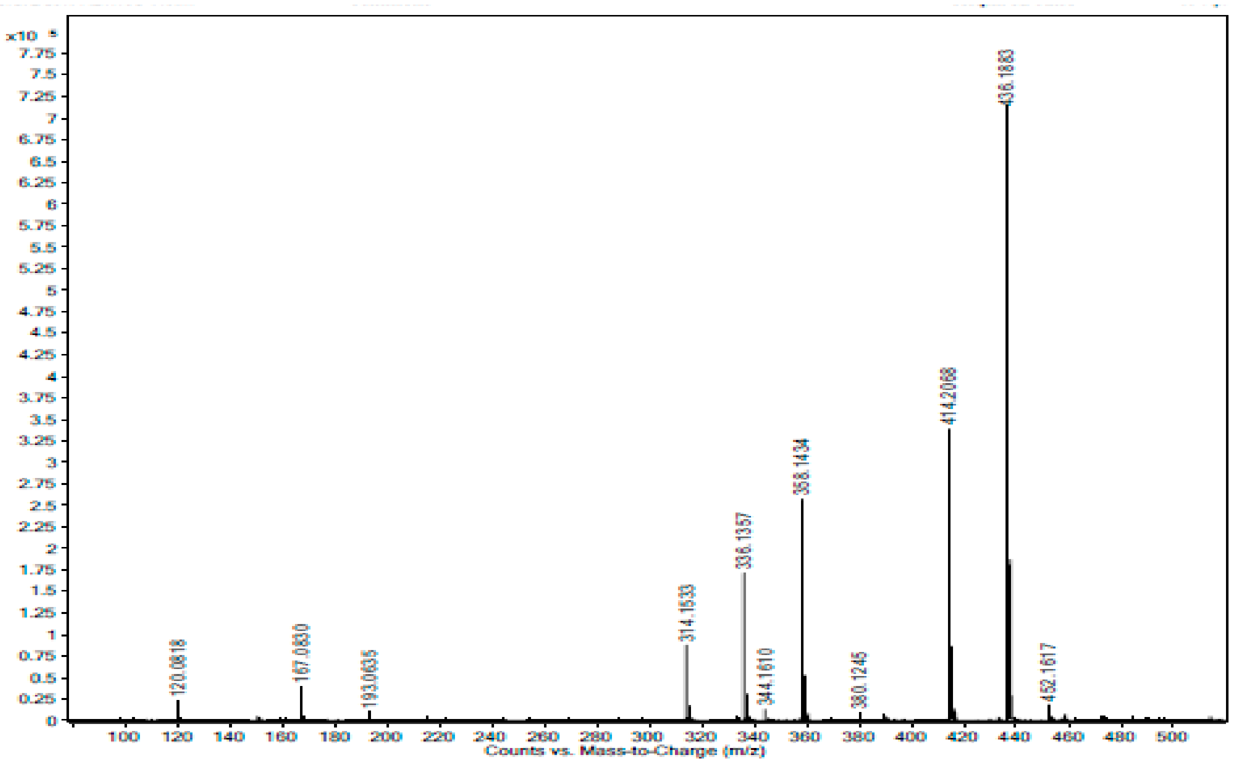

Figure S15. HRMS (ESI$^+$) of compound **4e.**

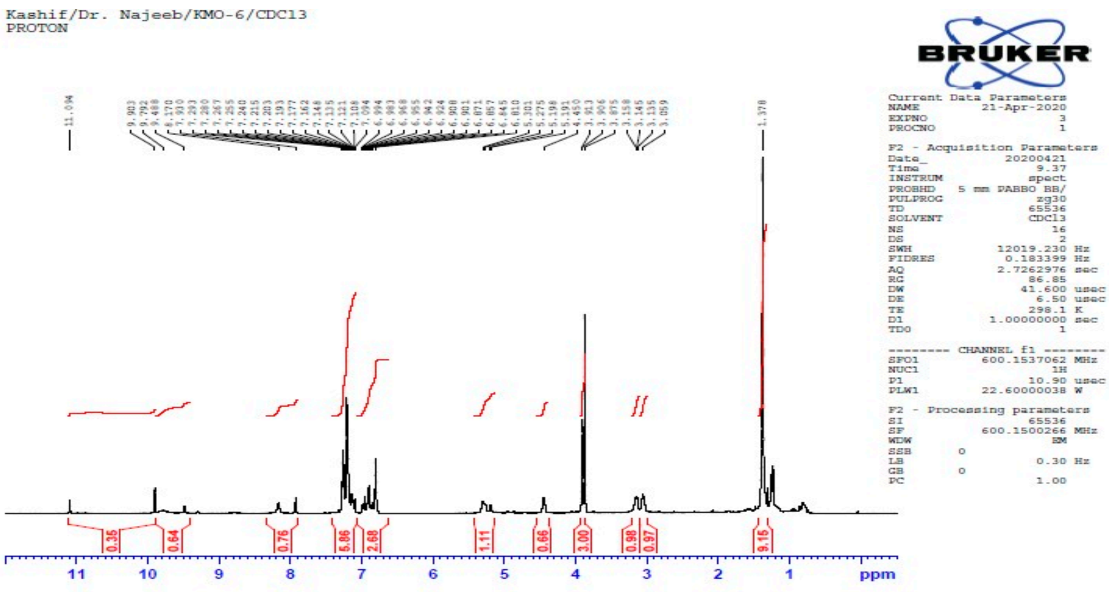

Figure S16. ^{1}H-NMR ($CDCl_3$, 600 MHz) of compound **4f.**

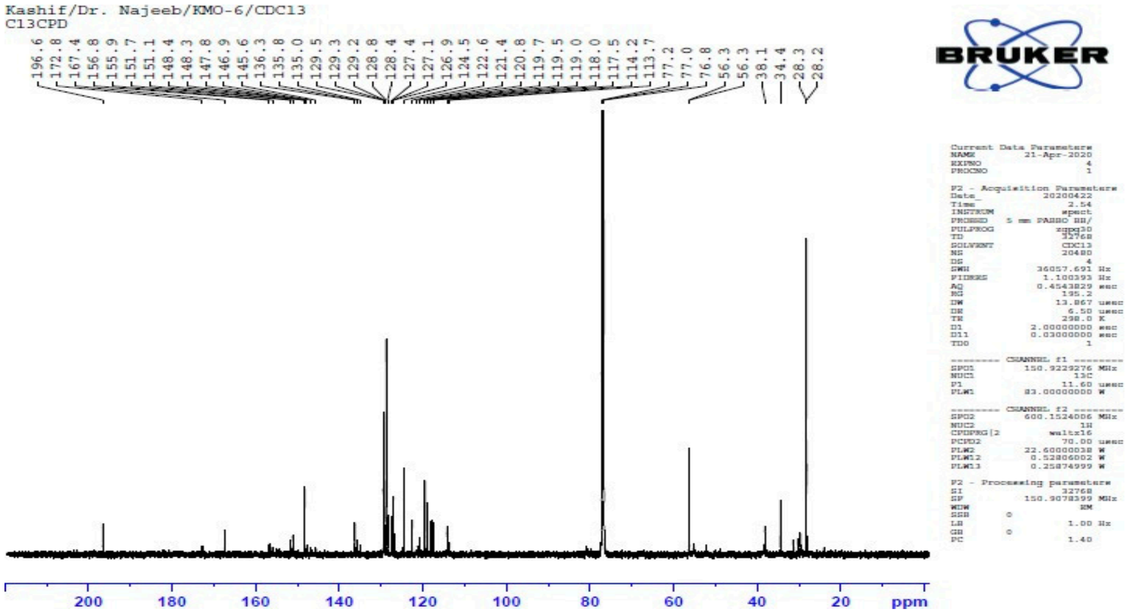

Figure S17. ^{13}C-NMR ($CDCl_3$, 125 MHz) of compound **4f.**

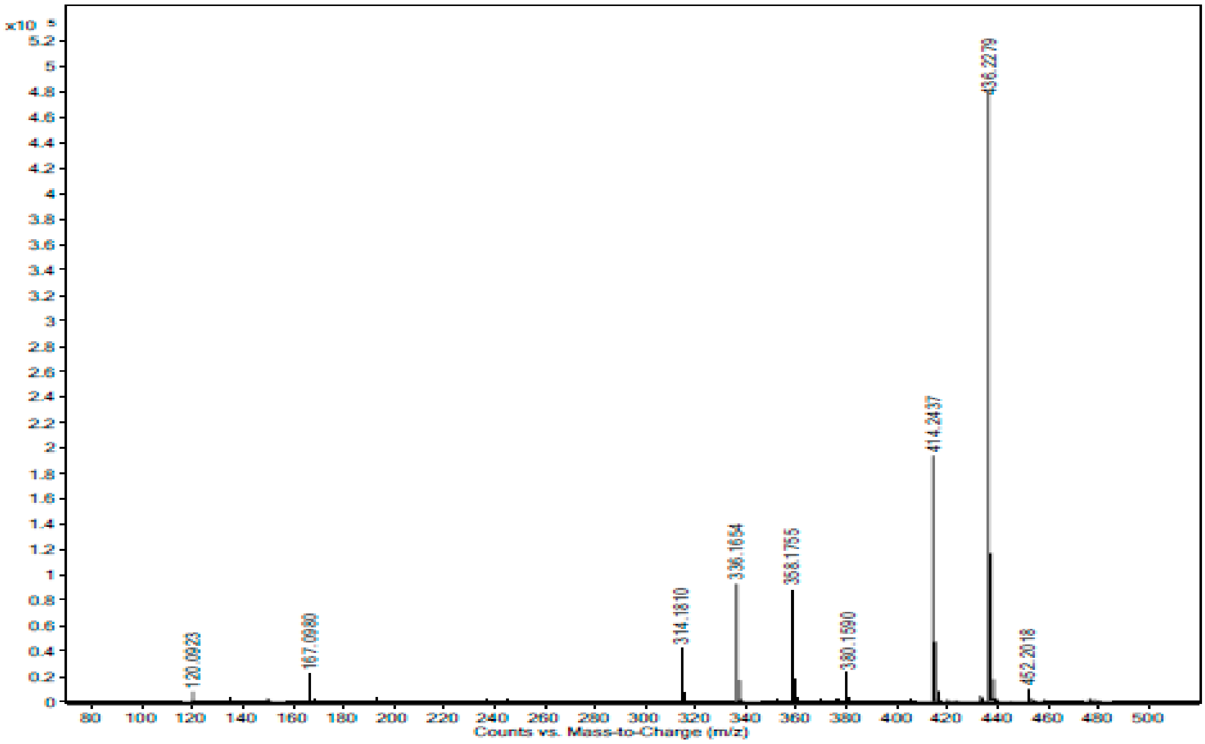

Figure S18. HRMS (ESI+) of compound **4f.**

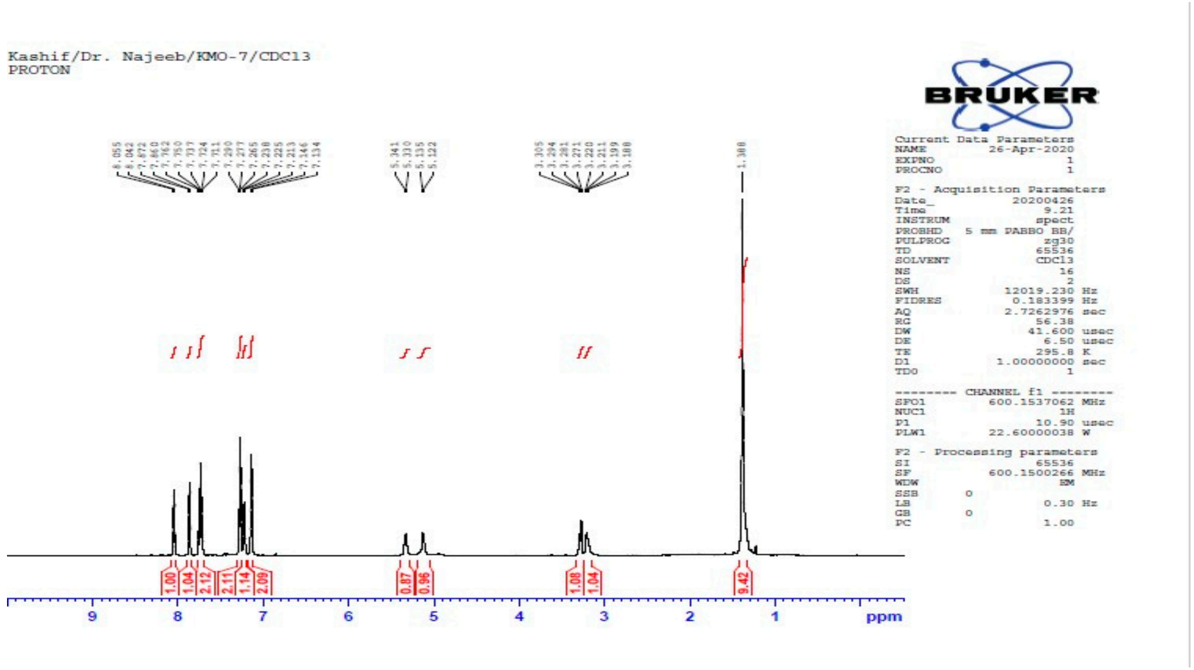

Figure S19. ^{1}H-NMR (CDCl3, 600 MHz) of compound **4g.**

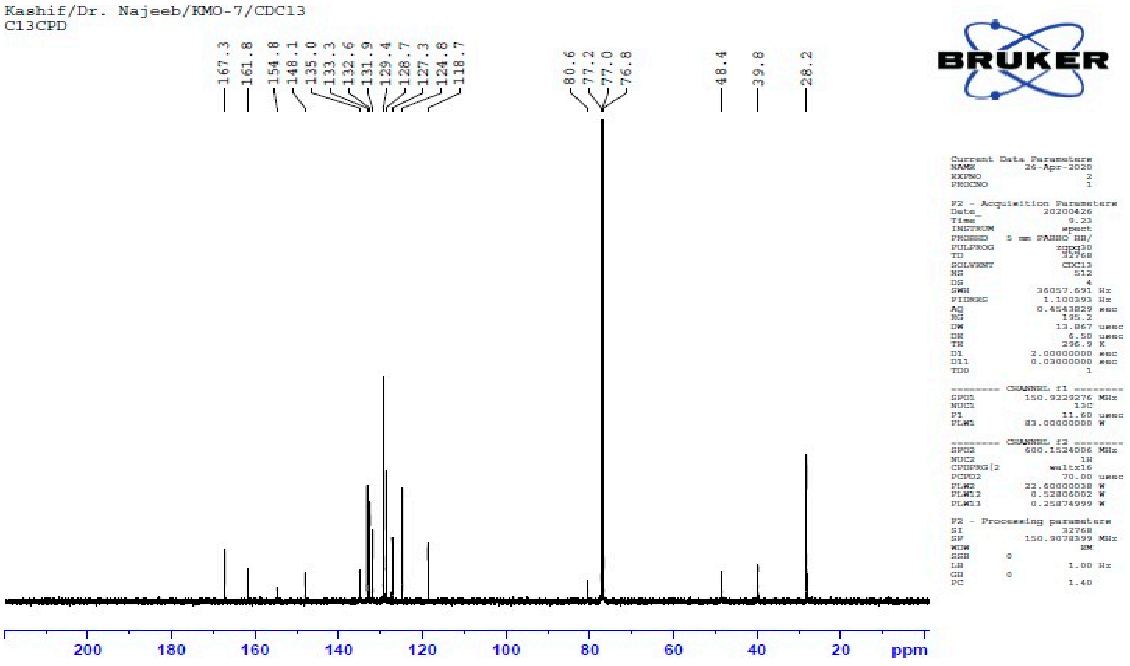

Figure S20. ^{13}C-NMR (CDCl3, 125 MHz) of compound **4g.**

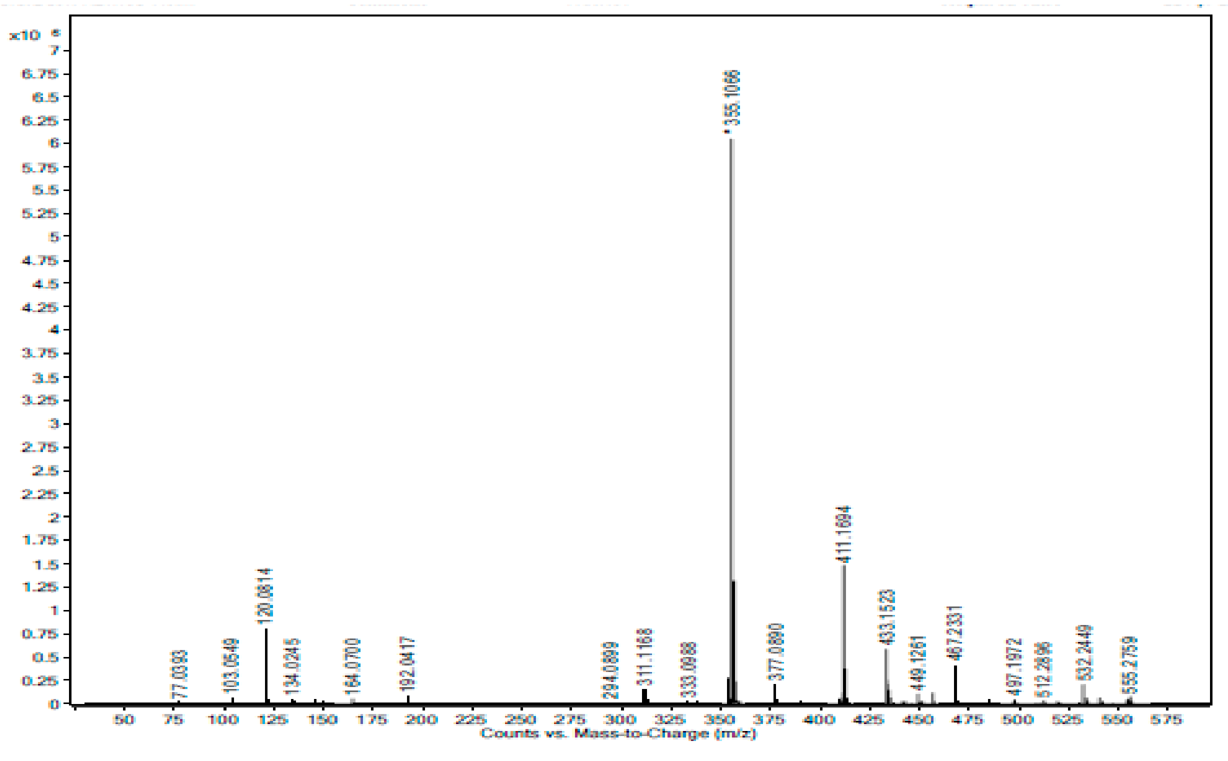

Figure S21. HRMS (ESI^{+}) of compound **4g.**

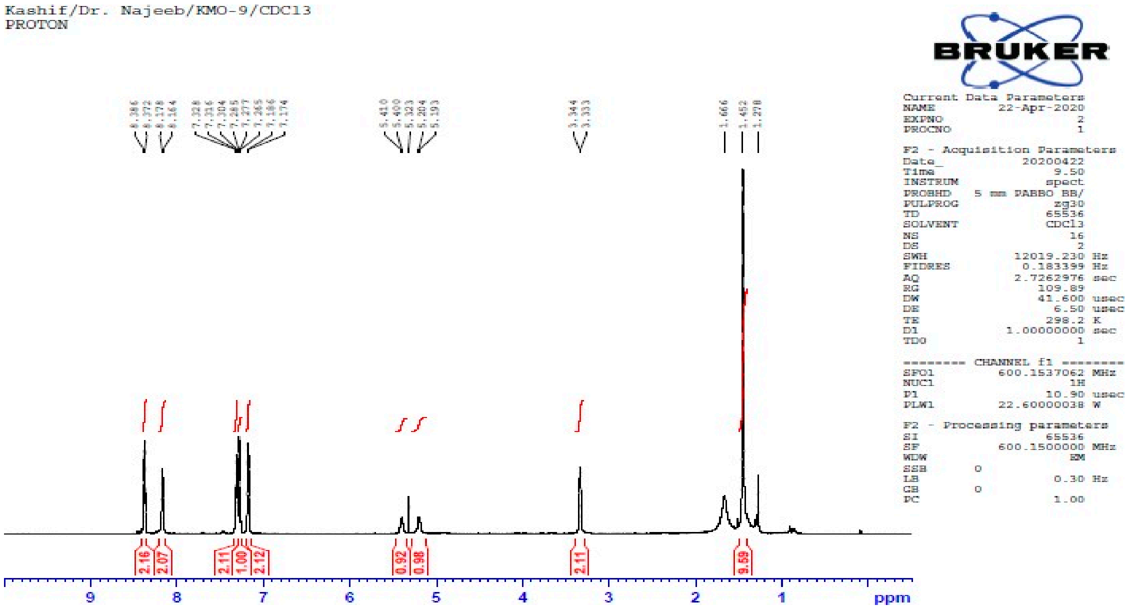

Figure S22. ^{1}H-NMR ($CDCl_3$, 600 MHz) of compound **4h.**

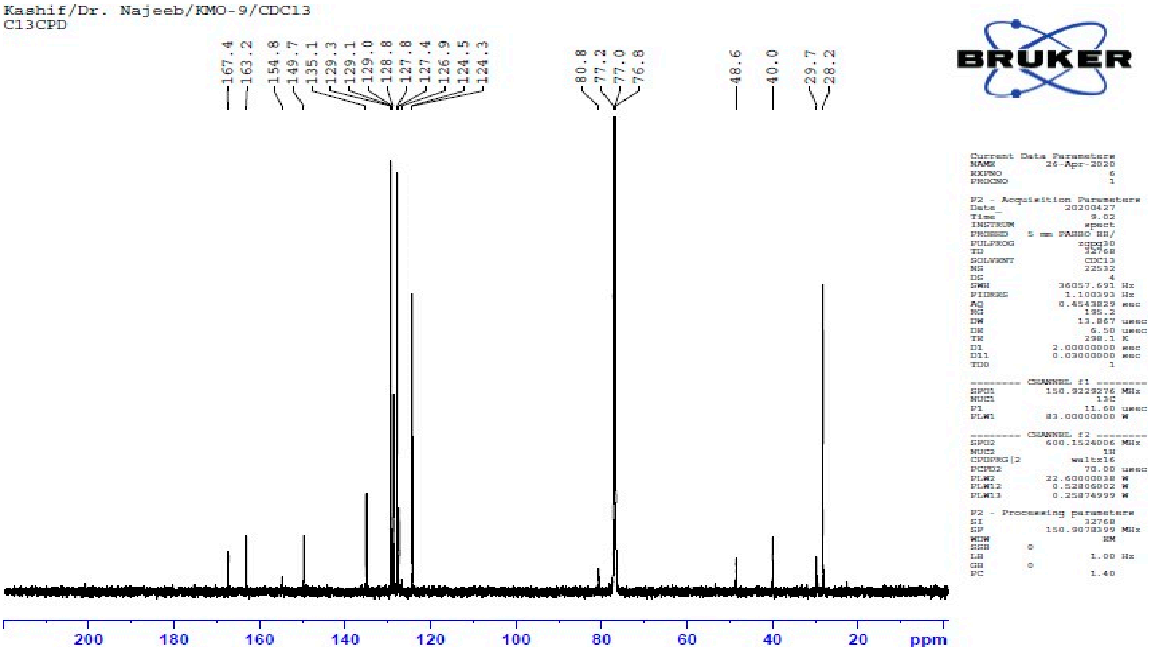

Figure S23. ^{13}C-NMR ($CDCl_3$, 125 MHz) of compound **4h.**

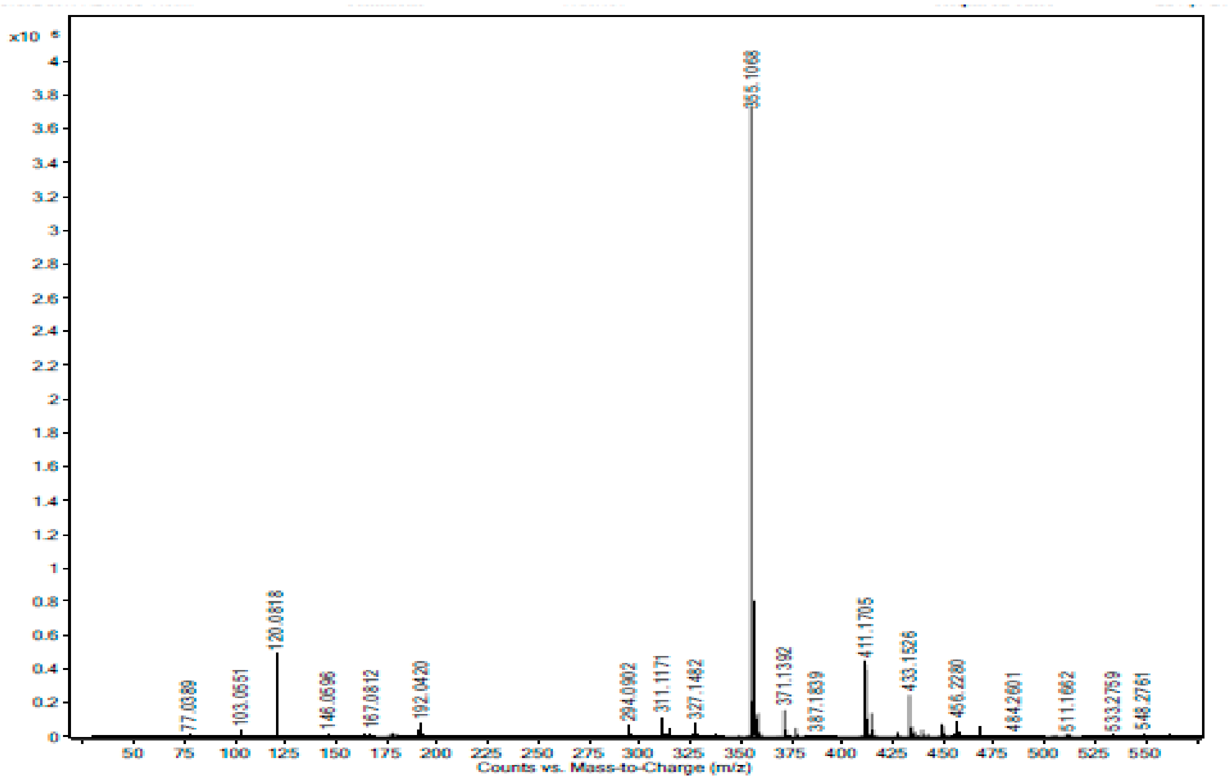

Figure S24. HRMS (ESI+) of compound **4h.**

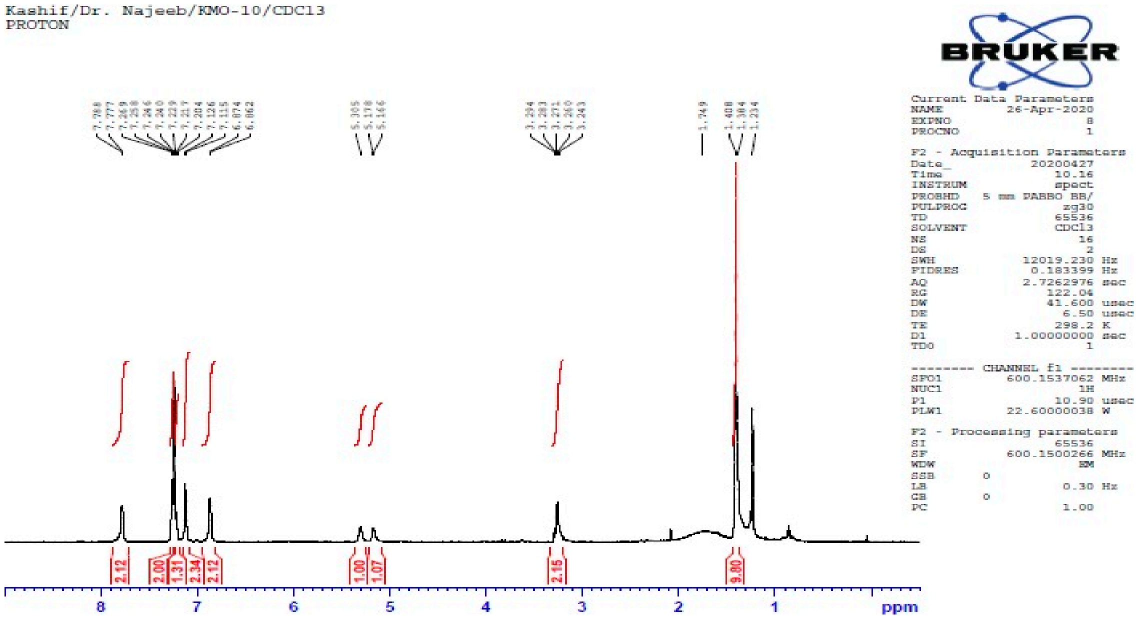

Figure S25. 1H-NMR (CDCl3, 600 MHz) of compound **4i.**

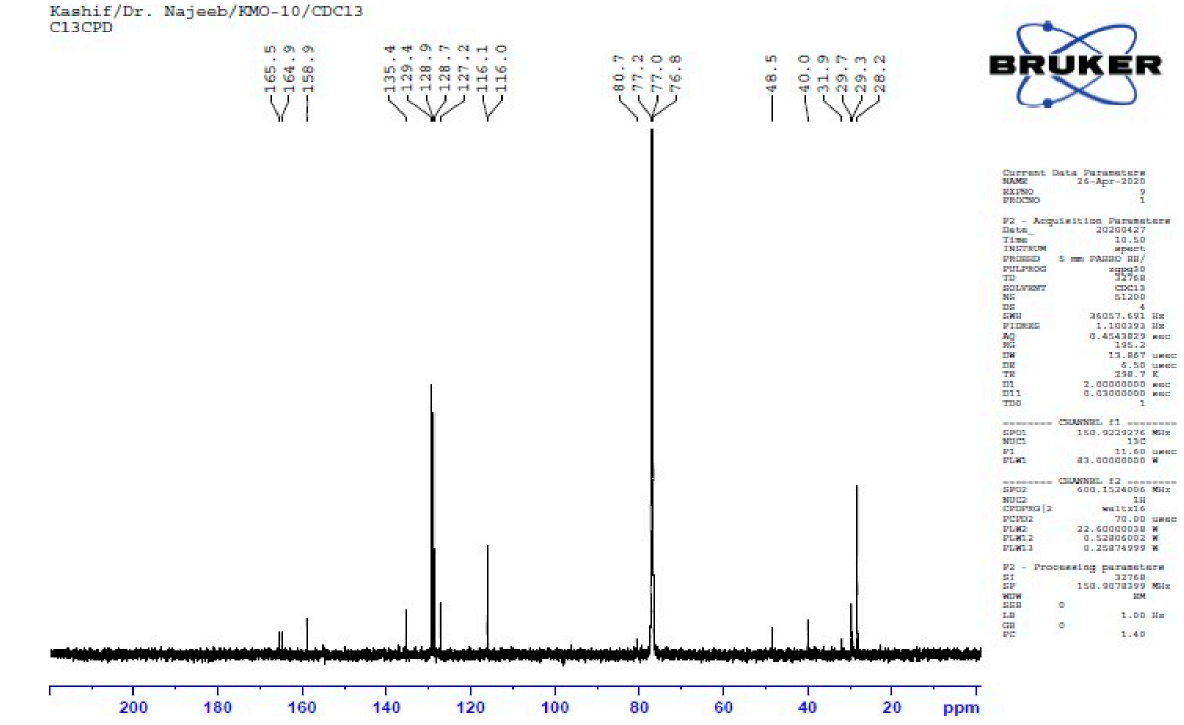

Figure S26. ^{13}C-NMR ($CDCl_3$, 125 MHz) of compound **4i.**

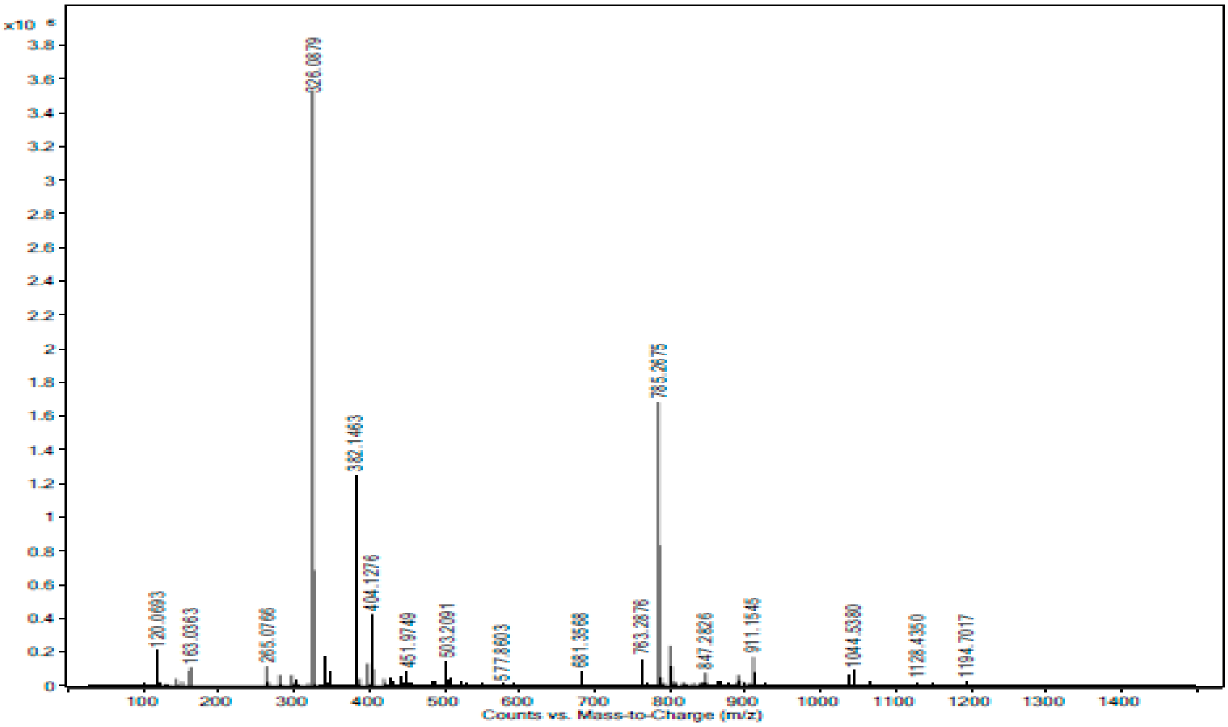

Figure S27. HRMS (ESI^+) of compound **4i.**

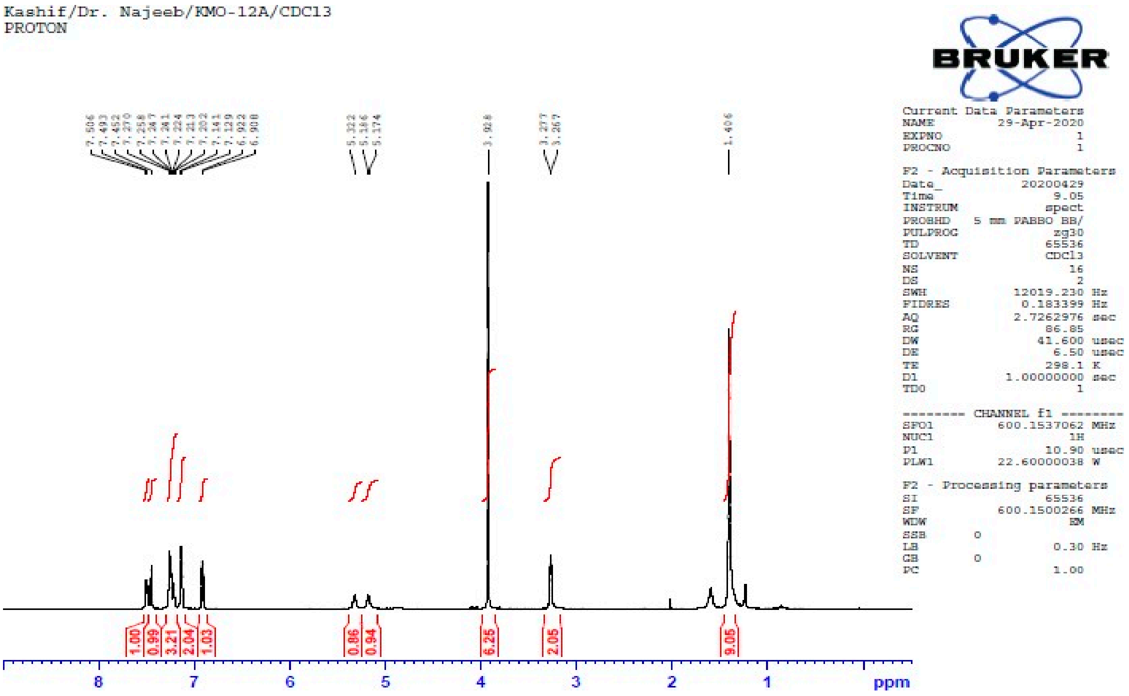

Figure S28. ^{1}H-NMR ($CDCl_3$, 600 MHz) of compound **4j.**

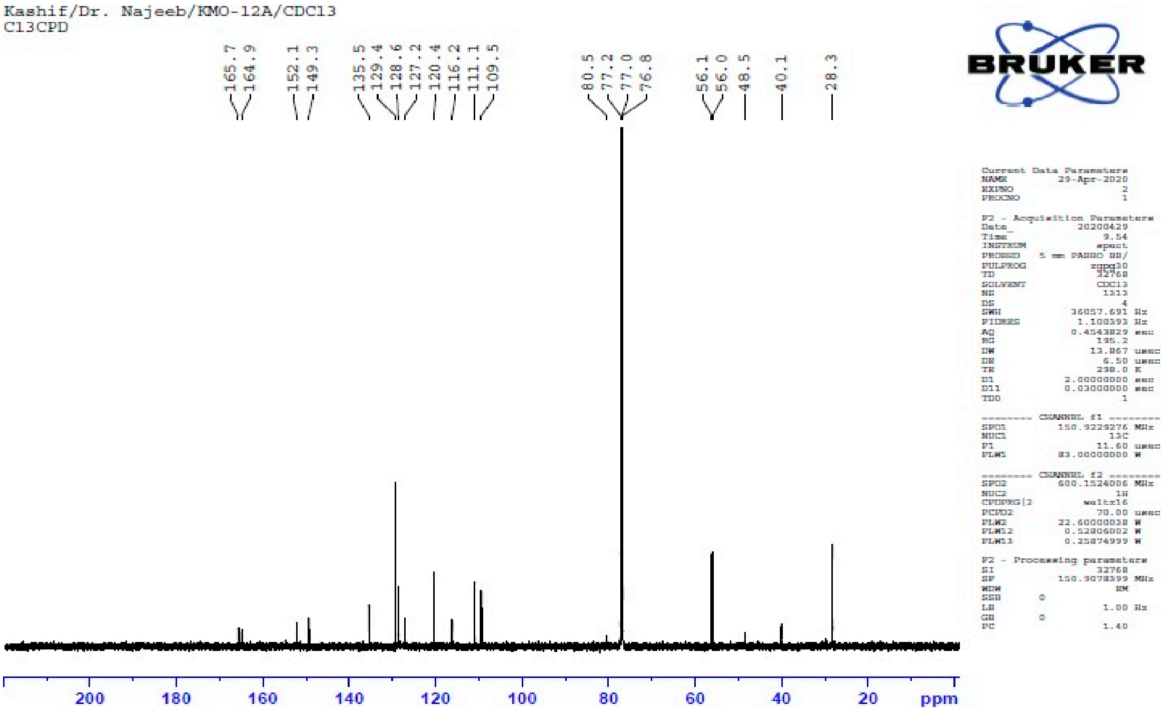

Figure S29. ^{13}C-NMR ($CDCl_3$, 125 MHz) of compound **4j.**

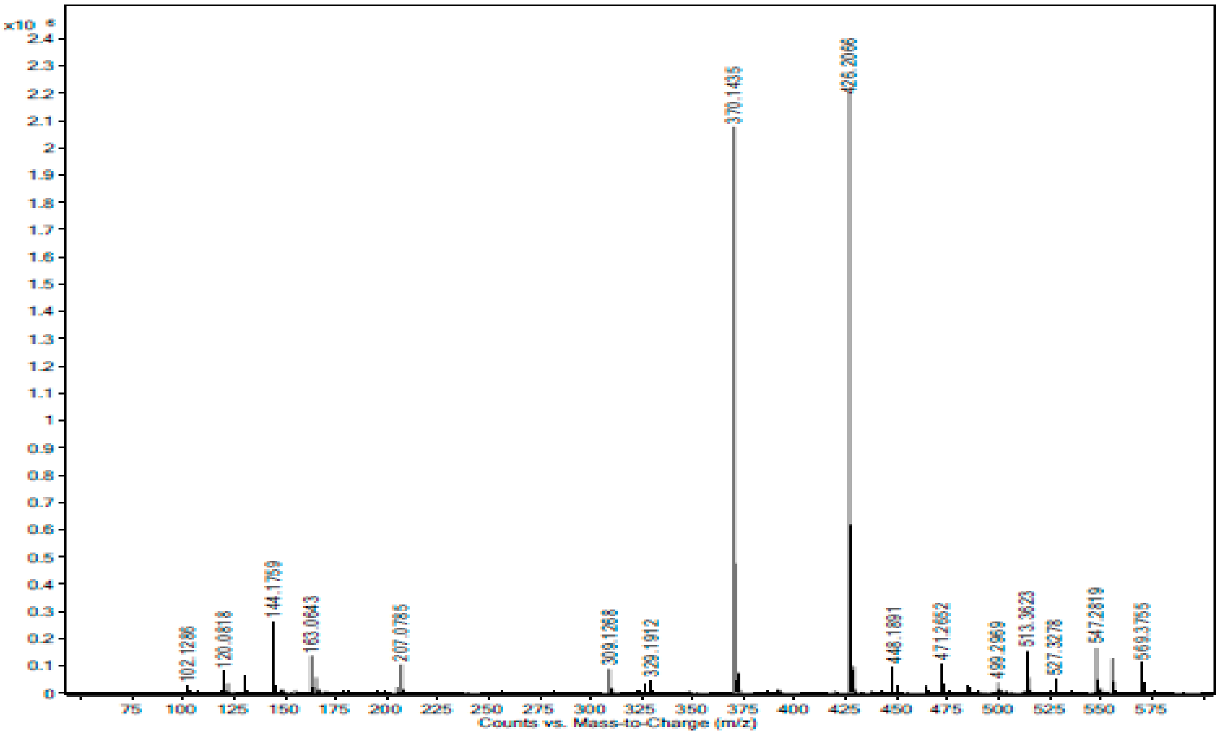

Figure S30. HRMS (ESI⁺) of compound **4j.**

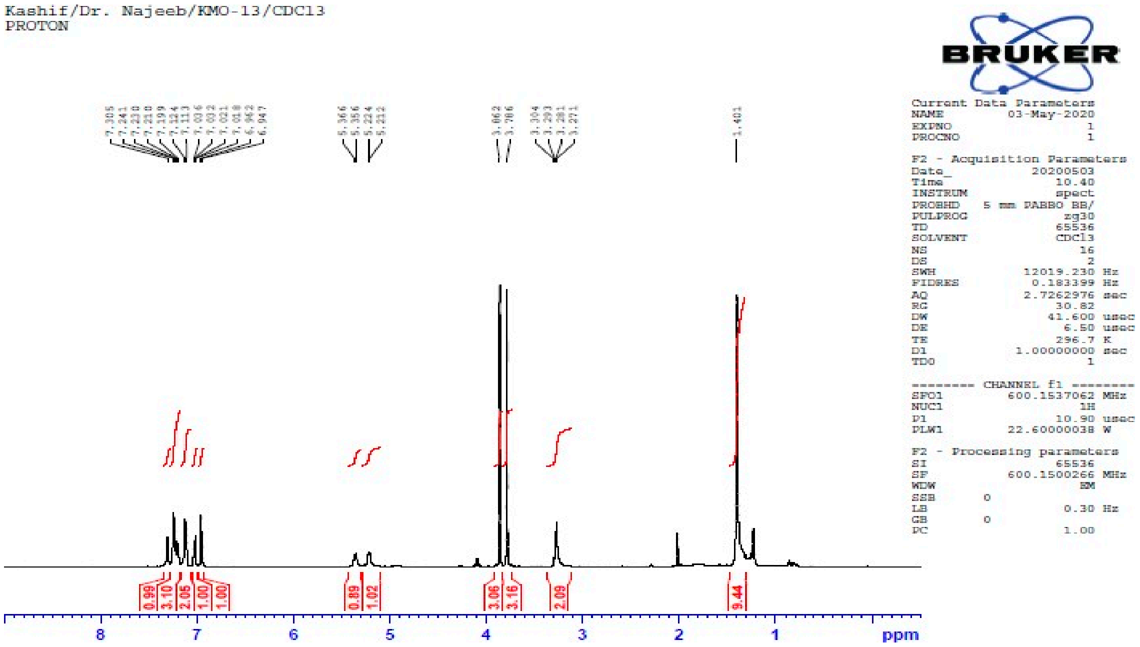

Figure S31. ¹H-NMR (CDCl3, 600 MHz) of compound **4k.**

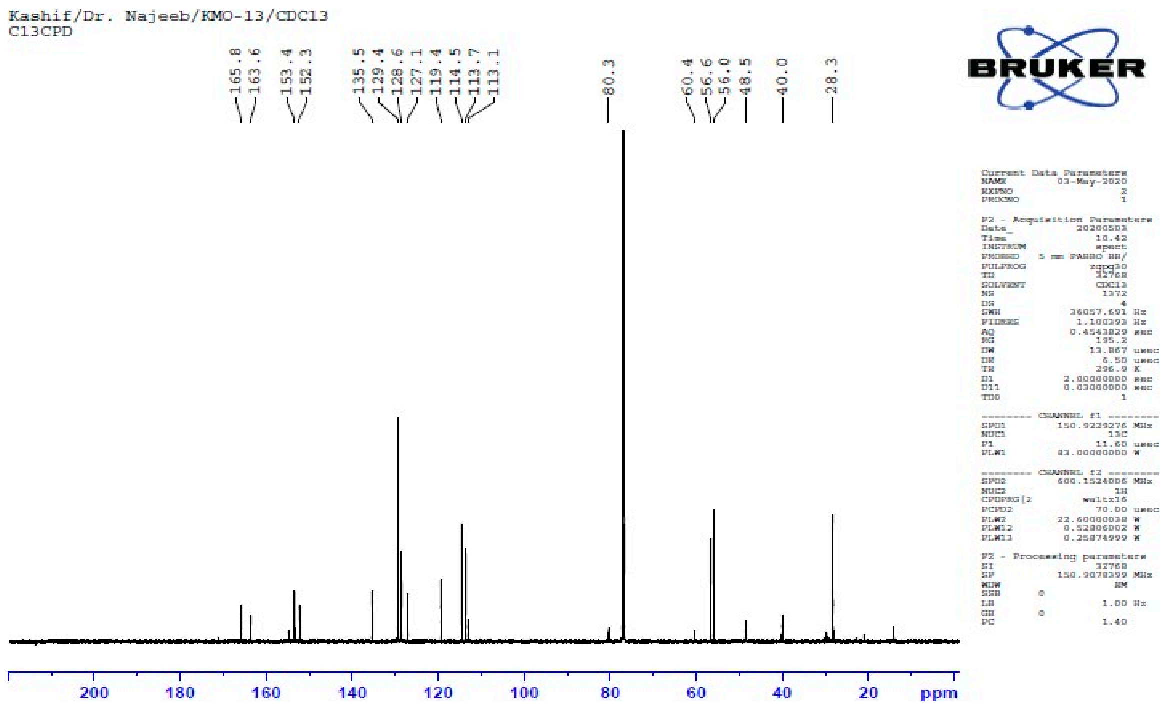

Figure S32. ^{13}C-NMR (CDCl3, 125 MHz) of compound **4k.**

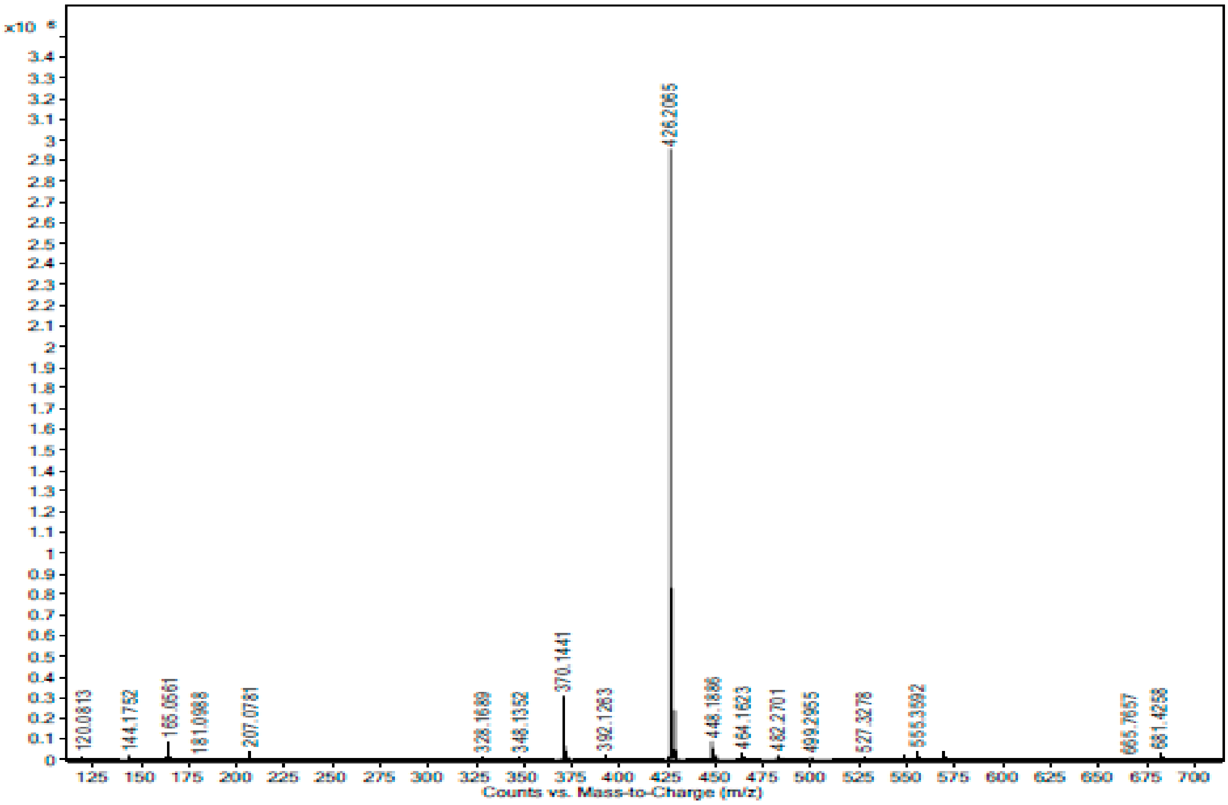

Figure S33. HRMS (ESI+) of compound **4k.**

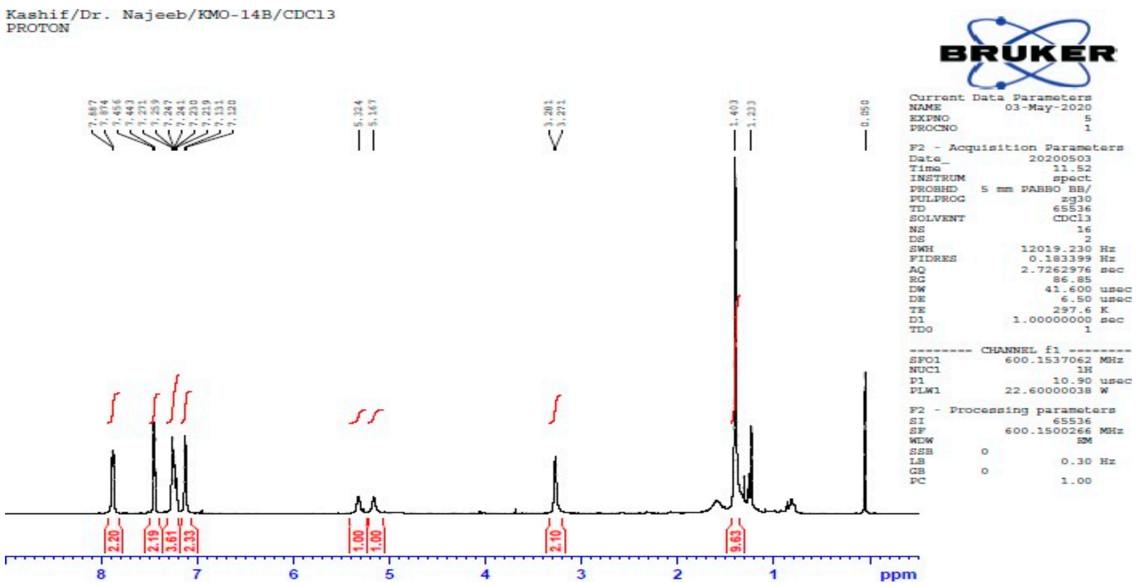

Figure S34. ¹H-NMR ($CDCl_3$, 600 MHz) of compound **4l.**

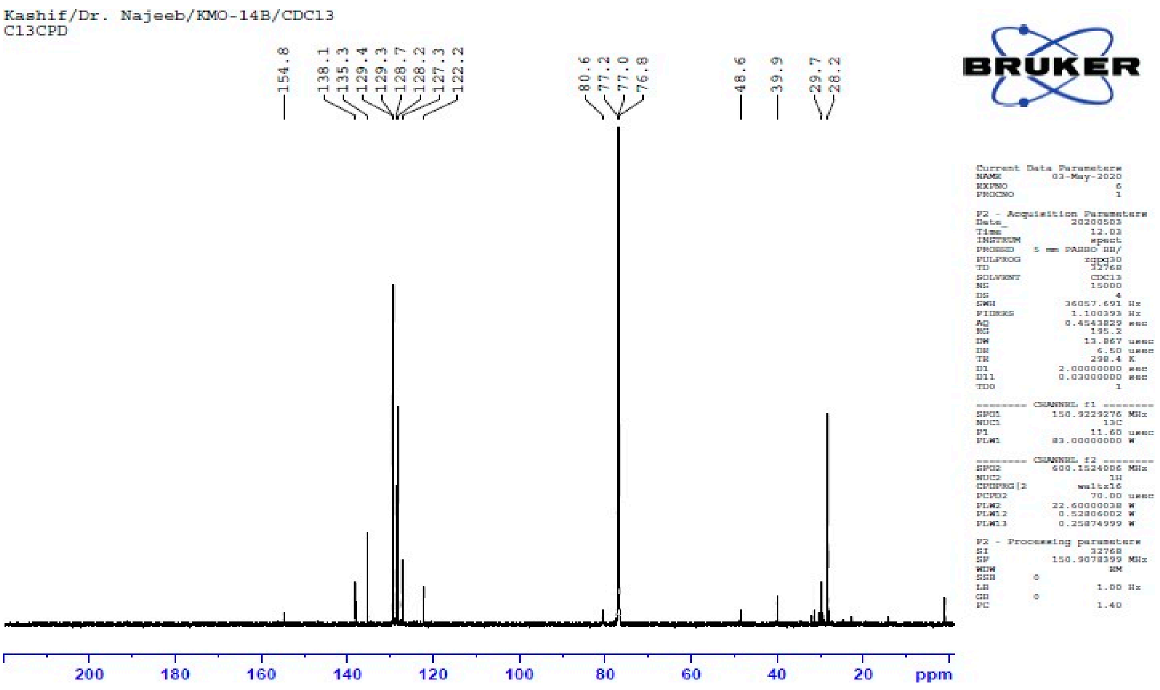

Figure S35. ¹³C-NMR ($CDCl_3$, 125 MHz) of compound **4l.**

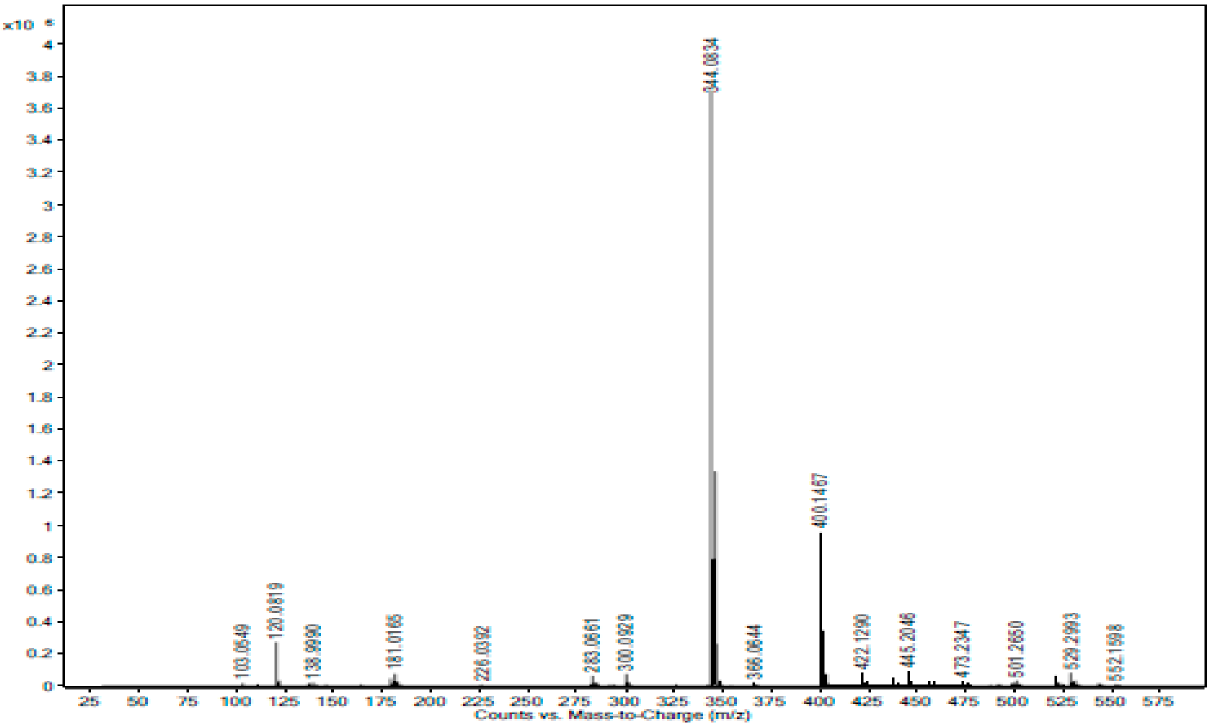

Figure S36. HRMS (ESI⁺) of compound **4l.**

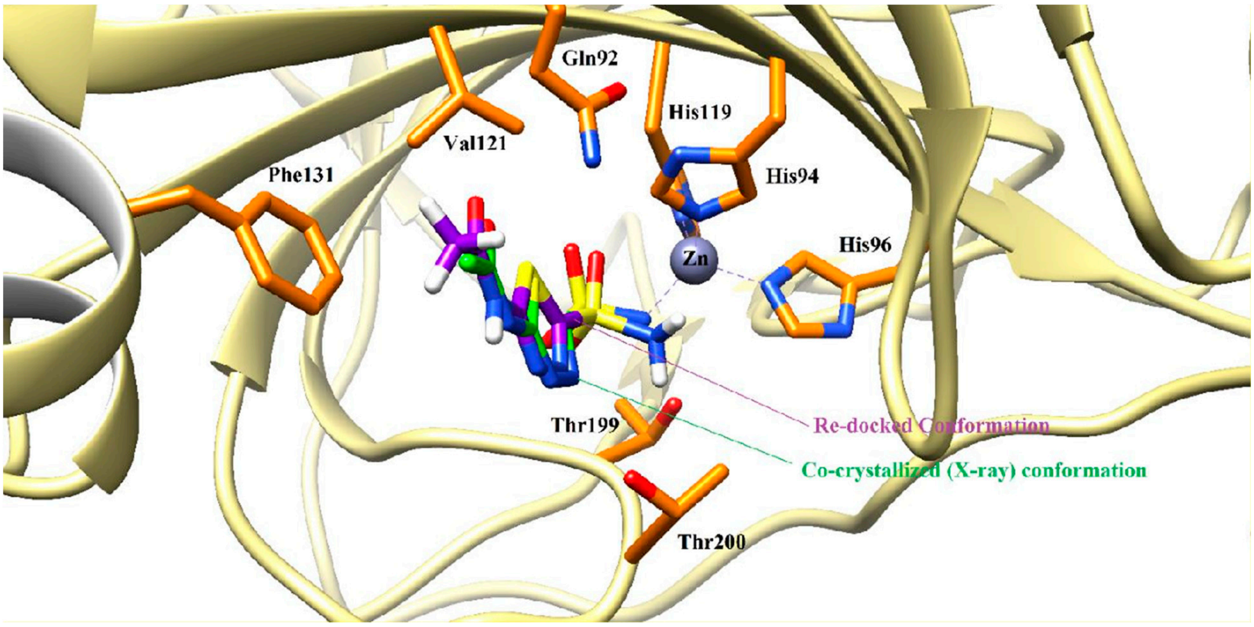

Figure S37. The superimposed view of re-docked orientation of acetazolamide with its reference conformation in the crystal structure (PDB code: 3HS4). The binding residues are shown in orange stick, protein is presented in ribbon model, the X-ray determined conformation and re-docked modes are shown in green and purple stick models, respectively.
